# Exploiting vitamin C as a prooxidant to activate ROS-responsive prodrugs for potent and selective tumor killing

**DOI:** 10.1016/j.redox.2026.104229

**Published:** 2026-06-04

**Authors:** Taufeeque Ali, Thilini Nimasha Fernando Ponnamperumage, Cody Joshua Miller, Daniel Li, Wasiu Olaniyi Awoyera, Alexis Kimberly Peterson, Hanlun Gao, Jatin Pandey, Julia Anna Rose Jakusz, Heli Fan, Gilbert Edward Koelsch, Leggy A. Arnold, Julie M. Jorns, Yee Chung Cheng, Avik Roy, Gang Zhou, Xiaohua Peng

**Affiliations:** a Department of Chemistry and Biochemistry and the Milwaukee Institute for Drug Discovery, University of Wisconsin-Milwaukee, 2000 E. Kenwood Boulevard, Milwaukee, WI, 53211, USA; b Department of Pathology, Medical College Wisconsin, Milwaukee, WI, USA; c Department of Hematology and Oncology and Department of Medicine, Medical College Wisconsin, Milwaukee, WI, USA; d Research and Development Laboratory, Simmaron Research Institute, Milwaukee, WI, USA; e Georgia Cancer Center, Medical College of Georgia, Augusta University, Augusta, GA, USA

**Keywords:** Combination therapy, Synergistic anticancer effects, ROS-Responsive prodrugs, Triple negative breast cancer, ROS-Generating agents, Vitamin C, Complete tumor regression

## Abstract

Developing targeted cancer therapy with minimal side effects remains a significant challenge. Oxidative stress-based cancer therapies have gained traction in recent years. However, challenges such as limited tumor selectivity and therapeutic durability often hinder their clinical application. Here, we report a novel strategy of combining ROS-responsive prodrugs with prooxidants to achieve potent, durable, and selective tumor killing effects. This approach leverages pro-oxidants (i.e. ascorbate) to amplify oxidative stress within tumors, sensitizing cancer cells to ROS-responsive prodrugs. Both *in vitro* and *in vivo* studies confirm the anticancer synergism and selectivity of this combination therapy, which achieved complete tumor regression without recurrence, significantly outperforming single-agent treatments. This combination therapy is effective against hard-to-treat cancers like triple-negative breast cancers. Our findings highlight the potential of targeting tumor redox mechanisms through a combination of ROS-responsive prodrugs and pro-oxidants, offering a promising avenue for repurposing these agents in cancer therapy.

## 1 Introduction

Cancer cells exhibit elevated oxidative stress due to oncogene activation, tumor suppressor gene inactivation, increased metabolism, and diminished antioxidant activities [[1], [2], [3], [4]]. Consequently, cancer cells tend to have higher basal level of reactive oxygen species (ROS) than normal cells. This feature has been exploited to develop therapeutic strategies that selectively target cancer cells while sparing normal tissues. One such strategy, known as oxidative stress-based cancer therapy, involves the use of pro-oxidants to drive ROS production and accumulation to an excessive level in cancer cells, resulting in irreversible damage to DNA, proteins and lipids that lead to eventual cell death. Numerous pro-oxidants have been identified for their ability to induce oxidative stress in cancer cells, showing promising results in certain cases [[2], [3], [4], [5], [6], [7], [8], [9], [10], [11]]. However, the application of oxidative stress-inducing pro-oxidants for cancer treatment faces many challenges, including limited tumor-selectivity, dose-limiting toxicity, inefficient drug delivery, acquired resistance, etc.

**Fig. 1:**
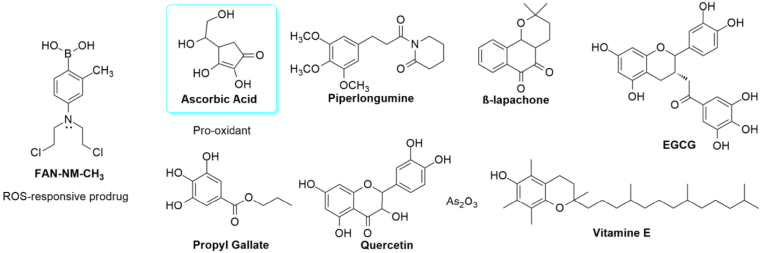
**ROS-responsive prodrug and pro-oxidants tested in combination therapy**.

The feature that cancer cells have elevated levels of ROS compared to normal cells has also been employed to develop a class of prodrugs that only become cytotoxic in the presence of ROS such as hydrogen peroxide (H_2_O_2_) [[12], [13], [14], [15], [16], [17], [18], [19], [20], [21], [22], [23]]. We have previously developed a group of prodrugs by coupling DNA alkylating agents with arylboronate or boronic acid, which can be activated by high H_2_O_2_ levels in cancer cells [17,18,[24], [25], [26]]. These compounds remain largely inactive until entering cancer cells, where elevated H_2_O_2_ levels convert them into potent alkylating agents. We have also demonstrated that these boronated prodrugs can indeed be activated in ROS-rich cellular compartments *in vitro* and *in vivo* by using ROS-responsive theranostic probes and deuterium-labeled prodrugs [17,27,28]. Among various ROS-activated DNA alkylating prodrugs, **FAN-NM-CH_3_** has emerged as a promising candidate with favorable drug-like properties and *in vivo* efficacy and selectivity [25]. (Fig. 1) Although ROS-activated prodrugs offer the potential to improve tumor selectivity and reduce systemic toxicity, their use as single-agent therapies faces several limitations, including intratumoral heterogeneity in ROS levels, insufficient prodrug activation, and limited durability of therapeutic responses. Strategies that enhance ROS-dependent activation may therefore be required to fully realize the therapeutic potential of ROS-activated prodrugs.

Here, we investigated whether combining ROS-activated prodrugs with pro-oxidants could enhance ROS-dependent prodrug activation and thereby achieve more selective, potent, and durable tumor killing. This strategy is based on the rationale that the ROS-amplifying effect of a pro-oxidant can fully activate a ROS-responsive prodrug in cancer cells to mediate tumor destruction more effectively than either drug alone. Through screening a panel of compounds with pro-oxidant activities, we identified vitamin C (**vitC**, ascorbate) as an effective partner for our lead ROS-responsive prodrug **FAN-NM-CH_3_.** The combination produced strong synergistic anticancer effects both *in vitro* and *in vivo* models. Mechanistic studies further revealed that the efficacy and tumor selectivity of this regimen depend on vitC-induced H_2_O_2_ production together with tumor-intrinsic deficiency in catalase activity, which limits the ability of cancer cells to detoxify ROS. Together, these findings establish a proof-of-concept therapeutic strategy in which pharmacological ROS amplification is harnessed to drive the activation of ROS-responsive prodrugs, thereby exploiting tumor redox vulnerability for selective cancer therapy.

## 2 Materials and methods

### 2.1 Cell culture

The human tumor cell lines MDA-MB-468 (HTB-132), MCF7 (HTB-22), MDA-MB-436 (HTB-130), MDA-MB-231 (HTB-26) and normal cell lines HMEC (PCS-600-010), MCF10A (CRL-10317) were purchased from the American Type Culture Collection. MDA-MB-468, MDA-MB-436, and MDA-MB-231 cells were cultured in L-15 Leibovitz media (Thermo Scientific Catalog: 41300070) supplemented with 10 % fetal bovine serum (FBS, Biowest: S1620), 1% non-essential amino acids (NEAA 100X solution, HyClone no: SH30238.01), and 1% penicillin and streptomycin (HyClone Penicillin Streptomycin 100X Solution, HyClone no: SV30010) at 37 °C in 100% relative humidity. MCF7 cells were maintained in ATCC-formulated Eagle's Minimum Essential Medium (30-2003) supplemented with 10 % fetal bovine serum (FBS, Biowest: S1620). 1% penicillin and streptomycin (HyClone Penicillin Streptomycin 100X Solution, HyClone no: SV30010) and 0.01 mg/mL human recombinant insulin (Sigma Aldrich Inc: 91077C) was added to MCF7 media. HMEC cells were maintained in Mammary Epithelial Cell Growth Media Kit (PCS-600-030, PCS-600-040) from ATCC. MCF10A cells were maintained in Lonza media kit MEGM (CC-3150) supplemented with 100 ng/mL cholera toxin. MCF7, HMEC, and MCF10A cells were maintained in 5% CO_2_ incubator at 37 °C.

### 2.2 Reagents and assay kits

The reagents used in this study includes Ascorbic Acid (VWR: BDH9242-100G), (−)-Epigallocatechin Gallate Hydrate (TCI America: 989-51-5), Piperlongumine (Indofine Chemical Co: 20069-09-4), Propyl Gallate (Mp Biomedicals Inc: 121-79-9), Quercetin (Asta Tech Inc: 117-39-5), Vitamin E (Santa Cruz Biotechnology: 10191-41-0), Arsenic (III) oxide (SIAL: 1327-53-3), β-Lapachone (Ambeed: 4707-32-8), Chlorambucil (Sigma-Aldrich: 305-03-3), Corning Matrigel (Corning: 354248), and Catalase (Sigma-Aldrich: C3155-100 MG). The Assay kits employed include Celltiter-Glo Reagent (Promega: G7570), Catalase Colorimetric Activity Kit (Invitrogen: EIACATC), Amplex Red Hydrogen Peroxide/Peroxidase Assay (Invitrogen, A22188), Dead Cell Apoptosis Kit with Annexin V Alexa Fluor 488 and Propidium Iodide (PI) (ThermoFisher Scientific: V-13245), DAPI solution (1 mg/mL) (ThermoFisher Scientific : 62248), Hydrogen Peroxide Assay Kit (Cell-based) (abcam: ab138874), Alkaline Comet Assay Kit (abcam: ab238544), IRDye 800CW 2-DG Optical Probe (LI-COR: 926-08946), and ROS Brite 700 probe (ATT Bioquest: 16004).

**Note:** For all *in vitro* treatments involving **vitC**, the cell culture media was replaced with serum-free Krebs-Ringer phosphate glucose (KRPG) buffer (145 mM NaCl, 5.7 mM sodium phosphate, 4.86 mM KCl, 0.54 mM CaCl_2_, 1.22 mM MgSO_4_, 5.5 mM glucose, pH 7.35) to prevent interference from serum components.

### 2.3 Cytotoxicity assay

General procedures for IC_50_ determination**.** Cells were plated into 96-well optical bottom plates (Nunc: 1256671) at densities ranging from 3000 to 5000 cells/well in 40 μL media and allowed to attach by incubation for 3 h before compound addition. **FAN-NM-CH**_**3**,_ Chlorambucil, Piperlongumine, β-Lapachone, Vitamin E, Propyl Gallate, Quercetin, and EGCG were solubilized in dimethyl sulfoxide (DMSO) at 20 mM stock. Arsenic Trioxide and **vitC** were dissolved in DI H_2_O (Arsenic stock: 20 mM; **vitC**: 1 M, pH adjusted to 7.0 with 1 M NaOH). All compounds were serially diluted 2-fold (11 dilutions). Using a Tecan Freedom EVO liquid handling system equipped with a 100 nL pin tool (V&P Scientific), 400 nL of each dilution was transferred to the cell plate (1:100 final dilution). All plates were incubated for an additional 48 h followed by the addition of 40 μL of Celltiter-Glo Reagent (Promega). Luminescence was measured after 30 min of incubation using an Infinite M1000 (Tecan) plate reader. Normalized % viability was assessed for each concentration against the individual vehicle treatments (DMSO or DI H_2_O).

IC_50_ determination of **FAN-NM-CH_3_** in combination with prooxidants. Pro-oxidants were prepared as follows: Piperlongumine (312 μM), β-Lapachone (312 μM), Vitamin E (624 μM), Propyl Gallate (10 mM), Quercetin (1.25 mM), and EGCG (5 mM) in DMSO; arsenic trioxide (316 μM) and **vitC** (400 mM, pH adjusted to 7.0 with 1 M NaOH) in DI H_2_O. A 100 nL aliquot of each stock was added to the assay plates (1:400 final dilution) 1 h prior to **FAN-NM-CH_3_** treatment. **FAN-NM-CH_3_** (20 mM DMSO stock) was serially diluted 2-fold (11 dilutions), and 400 nL of each dilution was dispensed to the same plates (1:100 final dilution). Cells were incubated for 48 h prior to viability analysis.

Effect of catalase on cytotoxicity. Catalase (7 μM) (Sigma-Aldrich C3155-100 MG) was diluted 10-fold in Milli-Q water, and 4 μL of the diluted enzyme was added to each well (1:10 final dilution). Plates were incubated for 1 h prior to **vitC** treatment. For vitC exposure, culture media was replaced with serum-free KRPG buffer. MDA-MB-468, MCF7, MCF10A, and HMEC received 100 nL (1:400 dilution) of **vitC** stocks at 400 mM, 100 mM, 400 mM, and 60 mM stocks (pH 7 in DI H_2_O), respectively, followed by 1 h incubation prior to **FAN-NM-CH_3_** addition. **FAN-NM-CH_3_** was added at 100 nL per well from 400 μM (MDA-MB-468), 800 μM (MCF7), 400 μM (MCF10A), 400 μM (HMEC) stocks in DMSO. PEG-conjugated catalase (PEG-CAT, Sigma-Aldrich C4963) was also tested to evaluate intracellular ROS scavenging. PEG-CAT produced effects consistent with standard catalase; due to its substantially higher cost, standard catalase was used for most experiments.

Effect of Deferoxamine (DFO). Cells *(*MDA-MB-468 cells, MCF7, MCF10A, and HMECs) were pre-treated with 100 μM DFO for 3 h, followed by treatment with **vitC** in serum-free KRPG buffer for 1 h, either alone or in combination with **FAN-NM-CH_3_** added in fresh media. Cytotoxicity was then assessed as described above.

### 2.4 Measurement of intracellular redox-active Fe^2+^ using BioTracker Far-Red Labile Fe^2+^ dye and flow cytometry

To measure changes in the redox-active iron pool (labile iron pool, LIP) in cultured cells, BioTracker Far-Red Labile Fe^2+^ Dye was used according to the manufacturer's instructions. This probe localizes to the cytoplasm, including the endoplasmic reticulum and Golgi apparatus, enabling detection of intracellular labile Fe^2+^. BioTracker Far-Red was purchased from Millipore Sigma (Cat. #SCT037). MDA-MB-468, MCF7, and MCF10A cells were seeded in 12-well plates and allowed to reach approximately 80% confluence prior to treatment. Cells were exposed to **vitC** in serum-free KRPG buffer for 1 h at concentrations of 1 mM for MDA-MB-468, 0.25 mM for MCF7, and 1 mM for MCF10A cells. Following **vitC** exposure, cells were treated with **FAN-NM-CH_3_** in fresh complete medium for 48 h at concentrations of 1 μM for MDA-MB-468, 2 μM for MCF7, and 1 μM for MCF10A cells, either alone or in combination with **vitC**. For mechanistic studies, cells were pretreated with deferoxamine (DFO, 100 μM) for 3 h or catalase (200 U/mL) for 1 h prior to **vitC** exposure and subsequent combination treatment. As assay controls, cells were treated with DFO (100 μM, 3 h) as a negative control to reduce the labile iron pool, whereas ferrous ammonium sulfate (FAS, 100 μM, 3 h) was used as a positive control for iron loading. Following treatment, cells were gently washed once with Hank's Balanced Salt Solution (HBSS; Ca^2+^/Mg^2+^ containing, phenol red-free). A 1 mM stock solution of BioTracker Far-Red dye was prepared in DMSO and diluted in HBSS to a final concentration of 5 μM. Cells were incubated with the staining solution at 37 °C for 1 h. After staining, cells were washed once with HBSS and detached using 0.25% trypsin. Trypsinization was quenched with HBSS, and cells were collected and maintained on ice. The cell suspension was centrifuged at 1200 rpm for 5 min, the supernatant was discarded, and cells were resuspended in PBS. Cells were washed once more with PBS, centrifuged again, and finally resuspended in PBS. Prior to acquisition, samples were passed through a 40 μm nylon mesh cell strainer to remove aggregates. Samples were kept on ice and analyzed immediately using a BD Accuri C6 flow cytometer. A minimum of 10,000 events per sample were collected using excitation at 635 nm and emission detection at 660 nm (FL4 channel). For analysis, cells were first gated using forward scatter (FSC-A) and side scatter (SSC-A) to exclude debris and select the live cell population. Singlets were subsequently gated by selecting the diagonal population on FSC-A versus FSC-H plots. Labile Fe^2+^ levels were quantified as the mean fluorescence intensity (MFI) of the BioTracker Far-Red signal in the FL4 channel from the gated live singlet population. Data were normalized to the indicated control conditions.

### 2.5 Long-term proliferative potential by clonogenic survival assay

Cells (1-2 × 10^5^) were plated in 6-well plates and allowed to adhere and grow in complete media for 48 h prior to treatment. For combination therapy, each well received 4 μL of a 750 mM stock of **vitC** in serum free KRPG buffer for 1 h, followed by 48 h of 4 μL of a 0.75 mM stock of **FAN-NM-CH_3_** in fresh media after replacing the buffer. In catalase experiments, 4 μL of a diluted catalase (1:10) was added 1 h before **vitC** exposure, whereas in iron chelation experiments, each well received 4 μL of a 75 mM stock of deferoxamine (DFO) 3 h prior to **vitC** treatment to ensure effective binding of labile metal ions. After treatment, cells were washed with PBS, trypsinized, and counted. An experimentally determined number of cells (1000 per well) were replated into fresh 6-well plates and incubated for 7–12 days to allow colony formation. Colonies were fixed with 70% ethanol for 20 min at room temperature, air-dried, and stained with Brilliant Blue methanol solution for 30 min. Excess stains were removed, wells were rinsed with tap water, and plates were dried overnight. Colonies containing more than 50 cells were counted, and plating efficiency was calculated for each treatment group.

### 2.6 Determination of combination index and dose reduction index

The Chau-Talalay interaction combination index (CI) and dose reduction index (DRI) [29,30] commonly used to determine the synergistic effect between two drugs, were calculated as follows:

$$CI=\left(\frac{D1}{Dx1}\right)+\left(\frac{D2}{Dx2}\right)$$

$$DRI\;1=\left(\frac{Dx1}{D1}\right)$$

$$DRI\;2=\left(\frac{Dx2}{D2}\right)$$

where Dx1 and Dx2 represent the doses of **FAN-NM-CH_3_** and **vitC** required to inhibit cell growth by 50%, respectively, and D1 (**FAN-NM-CH_3_**) and D2 (**vitC**) indicate the individual doses of the two drugs required for 50% inhibition of cell growth when used in combination. The combined effects of the two drugs are indicated as follows:

CI<1(Cooperativeeffect),CI=1(additiveeffect),andCI>1(antagonisticeffect)

A higher DRI value indicates a reduced drug dosage is needed in combination to achieve the same efficacy as a single drug.

### 2.7 Synergy evaluation by SynergyFinder 3.0

Cell viability was measured following a 48-h treatment period using the CellTiter-Glo assay. The percentage of viable cells relative to untreated control cells was determined to assess the cytotoxic effects of **vitC**, **FAN-NM-CH_3_**, and their combinations. Pairs of drugs were tested in combination at five serially diluted concentrations with a sixth concentration serving as a control. **vitC** was tested at concentrations ranging from 0 μM to 4000 μM, and **FAN-NM-CH_3_** was tested at concentrations ranging from 0.5 μM to 5 μM. Each combination and individual drug treatment was performed in at least three independent experiments per cell line. An excel matrix sheet was prepared containing Drug 1 and Drug 2 data with their corresponding percentage of viable cells relative to the untreated control, formatted for input into SynergyFinder. The SynergyFinder 3.0 platform (https://synergyfinder.fimm.fi/) was used to calculate and visualize synergy scores [31]. The Bliss Independence model was primarily used to analyze the synergy as it evaluates drug interactions by assuming independent action of the drugs. Synergy scoring was visualized as 2D and 3D interaction surfaces over the dose matrix. The depth of color in the two-dimensional image and the height of the three-dimensional landscape indicated the degree of synergy, additivity, or antagonism among drug combinations.

### 2.8 Determination of catalase activity

Catalase activity was measured using the Catalase Colorimetric Activity Kit (Invitrogen: EIACATC) according to the manufacturer's instructions. Cells (1 × 10^5^) were seeded in each well of 6-well tissue culture plates (VWR: 10062-892). Once the cells reached 90% confluency (∼1 × 10^6^ cells), they were treated with either no treatment, vehicle, **vitC**, **FAN-NM-CH_3_**, or a combination of both. Plates were then incubated for 48 h prior to sample preparation. The media was removed, and cells were washed twice with 3 mL of ice-cold PBS (without Mg^2+^ and Ca^2+^). Cells were gently dislodged using a rubber policeman, collected in 1.5 mL microcentrifuge tubes, and sonicated in 1 mL of cold 1X assay buffer. The samples were then centrifuged at 10,000 x g for 15 min at 4 °C, and the collected supernatants were normalized to 100 μg/mL of protein using Pierce Detergent compatible Bradford assay reagent (Thermo Scientific: 23246S). The collected samples were diluted 1:5 in 1X assay buffer. Catalase standards were prepared by diluting 10 μL of the catalase standard solution in 190 μL of 1X assay buffer to obtain a 5 U/mL catalase solution. This solution was further diluted 2-fold five times, including a blank with 1X assay buffer. Fresh standards were prepared 1-2 h prior to use. 25 μL of the standards and diluted samples were added to the clear 96-well half-area plate provided with the kit, followed by 25 μL of Hydrogen Peroxide Reagent into each well. The plate was incubated for 30 min at room temperature. Subsequently, 25 μL of substrate and 25 μL of 1X HRP solution were added to each well. The plate was incubated at room temperature for 15 min. Absorbance was recorded at 560 nm using the Infinite M1000 (Tecan) plate reader. A standard curve was generated using curve-fitting on GraphPad Prism software, and the activity of unknown samples was calculated from the standard curve and adjusted for the appropriate dilution factor.

### 2.9 RNA extraction and RT-qPCR

Cells (1 × 10^5^) were seeded in each well of 6-well tissue culture plates (VWR: 10062-892). Once the cells reached 90% confluency (∼1 × 10^6^ cells), they were treated with either vehicle, **vitC**, **FAN-NM-CH_3_**, or a combination of both. Plates were then incubated for 48 h prior to sample preparation. Cells were harvested and resuspended in 350 μL of RLT buffer in the presence of 1% β-mercaptoethanol. QIAshredder spin columns (Qiagen: 79656) was used to lyse the cells and total isolated RNA was purified with RNAeasy kit (Qiagen: 74104). Quantification was done by reading absorbance (260 nm/280 nm) using Infinite M1000 (Tecan) plate reader. RNA was reverse transcribed into cDNA using qScript One-Step SYBR Green qRT-PCR Kit (QuantaBio: 95054-946). Primers used are as follows: GAPDH forward primer (FP) 5′-ACCACAGTCCATGCCATCAC-3′, GAPDH reverse primer (RP) 5′-TCCACCACCCTGTTGCTGTA-3′; P53 FP 5′-GTTCCGAGAGCT GAATGAG-3′, P53 RP 5′-TTATGGCGGGAGGTAGACTG-3’; Catalase FP 5′-CCAGAAGAAAGC GGTCAAGAA-3′, Catalase RP 5′-GAGATCCGGACTGCACAAAG-3’. cDNA synthesis and amplification were done on an Eppendorf Mastercycler in a 96-well twin.tec PCR plate (Eppendorf: 951022015). A 20 μL reaction volume comprised of 10 μL SYBRGreen Master Mix 2X, 4.8 μL RNase-Free water, 1 μL of each primer, 0.2 μL of RT, and 3 μL of RNA (50 ng) in each well. Melting curve was used to assess target specificity. The gene expressions were normalized to relative GAPDH expression. Relative expression of each gene against their controls were calculated using the 2^−ΔΔCt^ method of Livak and Schmittgen. Standard deviations were calculated for three biologically independent trials performed in triplicates.

### 2.10 Measurement of extracellular/intracellular H_2_O_2_ levels

Extracellular H_2_O_2_ levels were determined using an Amplex Red Hydrogen Peroxide/Peroxidase Assay (Invitrogen: A22188) and intracellular H_2_O_2_ levels were determined using Hydrogen Peroxide Assay Kit (abcam: ab138874) as per their respective manufacturer's protocol. Briefly 25x10^3^-50 × 10^3^ cells were seeded in each well of a 96 Well Black, Optically Clear Polymer Bottom Plate (Thermo Scientific™ Catalog: 1256670) in 40 μL (Final reaction volume). The plates were incubated for 3 h prior to the addition of the compounds. *Ascorbic acid dose-dependent H*_*2*_*O*_*2*_ *detection:* **vitC** was dissolved in DI H_2_O at 1 M stock concentration and pH adjusted to 7.0 using 1 M Sodium Hydroxide solution. The stock was serially diluted (2-fold, 11 times). 400 nL of these serially diluted stocks were added to the cell plate (1:100 dilution) using a Tecan Freedom EVO liquid handling system equipped with a 100 nL pin tool (V&P Scientific). *Dose response:* A 7 μM Catalase solution (Sigma-Aldrich: C3155-100 MG) was diluted 10-fold in Millipore water. 4 μL of the diluted catalase solution was added to the cell plate (1:10). Plates were incubated for 1 h prior to the addition of **vitC**. MDA-MB-468, MCF7, MCF10A, and HMEC received 100 nL (1:400) of the 400 mM, 100 mM, 400 mM and 60 mM stocks of **vitC** in DI H_2_O respectively (**vitC** pH was adjusted to 7.0 with sodium hydroxide). Plates were then incubated for another hour prior to the addition of **FAN-NM-CH_3_**. 100 nL of 400 μM, 800 μM, 400 μM, and 400 μM stocks of **FAN-NM-CH_3_** in DMSO were added to each MDA-MB-468, MCF7, MCF10A, and HMEC assay plates respectively. All Plates were incubated for an additional 48 h. *Controls:* All controls were freshly prepared using ∼3% Hydrogen peroxide (comes with each kit) in 1X KRPG buffer (Krebs–Ringer phosphate consists of 145 mM NaCl, 5.7 mM sodium phosphate, 4.86 mM KCl, 0.54 mM CaCl_2_, 1.22 mM MgSO_4_, 5.5 mM glucose, pH 7.35) prior to assaying. *Extracellular H*_*2*_*O*_*2*_ *levels:* After 48 h incubation, the cells were washed twice with 1X KRPG buffer and incubated for 5 h in 40 μL KRPG buffer. Then, 20 μL of this KRPG buffer was transferred into another 96 Well Black, Optically Clear Polymer Bottom Plate (Thermo Scientific™ Catalog: 1256670) in triplicates and mixed with an equal amount of Amplex Red reagent (50 μM Amplex Red and 0.1 U/mL HRP final concentrations). After 5 h incubation, fluorescence was measured (Ex/Em: 560/590 nm) on an infinite M1000 (Tecan) microplate reader. Note that the final concentration of H_2_O_2_ controls has been adjusted to account for the dilution by the same volume of Amplex Red reagent. *Intracellular H*_*2*_*O*_*2*_ *levels:* After 48 h incubation, the cells were washed twice with 1X KRPG buffer. 40 μL of 1X AbGreen in assay buffer was added to each well. Plates were incubated for 60 min at room temperature in dark. Fluorescence was measured (Ex/Em: 490/520) on an infinite M1000 (Tecan) microplate reader. The same plates were used for fluorescence microscopic imaging on an EVOS Digital Inverted Microscope.

### 2.11 Detection of cell apoptosis

Apoptosis was assessed using Dead Cell Apoptosis Kit with Annexin V Alexa Fluor 488 with Propidium Iodide (PI) (ThermoFisher Scientific: V-13245), and DAPI solution (1 mg/mL) (ThermoFisher Scientific (62248) as per manufacturer's protocol. 50 × 10^3^ cells were seeded in each well of the Thermo Scientific Nunc Lab-Tek II chamber slide (Thermo Scientific: 125658) with a final reaction volume of 500 μL and incubated for 3 h prior to the treatment. MDA-MB-468, MCF7, and MCF10A received 1.25 μL (1:400) of the 400 mM, 100 mM, and 400 mM stocks of **vitC** in DI H_2_O respectively (**vitC** pH was adjusted to 7.0 with sodium hydroxide). Plates were then incubated for another hour prior to the addition of **FAN-NM-CH_3_**. 1.25 μL of 400 μM, 800 μM, and 400 μM stocks of **FAN-NM-CH_3_** in DMSO were added to each MDA-MB-468, MCF7, and MCF10A assay plates respectively. All Plates were incubated for an additional 48 h. After the treatment, cells were washed with ice-cold PBS twice. After the wash, 20 μL of the Annexin V and 2 μL of the 100 μg/mL PI stock in 78 μL 1X Annexin binding buffer was added to each well with a final reaction volume of 100 μL. The chamber slides were incubated for 30 min and washed with a 1X Annexin binding buffer. A final wash was done with 1:500 dilution of the 1 mg/mL DAPI solution in PBS for 5 min. Chambers were detached and slides were mounted with the Fluoromount (TM) Aqueous Mounting Medium (Sigma-Aldrich: F4680) with a glass cover slip. The slides were kept in the dark and imaged the next day in the dark room on an Accu Scope EXC-500 Fluorescence microscope system.

### 2.12 Alkaline comet assays

The comet assay was performed according to manufacturer's protocol (Abcam: ab238544). Cells were seeded in 6-well tissue culture plates (VWR: 10062-892) at a density of 1 × 10^5^ cells. Once cells reached 90% confluency, they were treated at varied conditions for 48 h. Cells were gently removed from the 6-well plate with a rubber policeman in 1 mL ice-cold PBS (without Mg^2+^ and Ca^2+^). Cells were isolated by centrifugation at 700 x g for 3 min. Supernatant was discarded. Cell pellet was washed again in ice-cold PBS. Finally, cells were resuspended in PBS and further diluted to 1 × 10^5^ cells/mL. 180 μL of the preheated comet agarose maintain at 37 °C in water bath was gently mixed with 20 μL cell samples (1:10). 150 μL/well of this mix was transferred onto a pre-warmed glass and maintained horizontally for 5 min. Slides were then transferred to 4 °C in the dark for 30 min to let the agarose solidify. The slides were then transferred into a small basin containing pre-chilled lysis buffer at 4 °C for 2 h in the dark. The Lysis buffer was then replaced with pre-chilled alkaline unwinding solution (300 mM NaOH, 1 mM EDTA) in 4 °C for 30 min in the dark. The slides were then gently transferred into a horizontal electrophoresis chamber with pre-chilled alkaline electrophoresis solution (300 mM NaOH, 1 mM EDTA, pH > 13) and ran with a voltage of 35 V applied for 25 min. Slides were then slowly removed and rinsed with DI water twice for 2 min followed by cold 70% ethanol for 5 min. The slides were allowed to air dry in the dark for 1 h. 100 μL/well of diluted Vista Green DNA dye was added onto the agarose. The slides were then incubated at room temperature for 15 min in the dark. Comets were analyzed under an EVOS Digital Inverted Microscope at 20X magnification. DNA damage was quantified using TriTek CometScore Software.

### 2.13 Experimental animals

Six-week-old female CD1 mice from Charles River Laboratory were used for a safety study, while immune-deficient female nude mice (Charles River Strain, Code 490) weighing 22−25 g were used for an *in vivo* efficacy study. The animals were housed under specific pathogen-free conditions, maintained under standard humidity, temperature, and a controlled 12-h light/dark cycle, with free access to food and water. All animals were allowed to acclimate for approximately seven days before experimental procedures. All animal experiments complied with the University of Wisconsin−Milwaukee Institutional Animal Care and Use Committee (IACUC) guidelines.

### 2.14 Safety study

The maximum tolerated dose (MTD), defined as the highest dose not causing a serious adverse event (e.g., death, convulsion, ataxia, aberrant behavior, or evident pain) observed within 2 d of observation, was determined for the prodrug and **vitC** among female CD1 mice using groups of three animals per group. **vitC** was dissolved in DI water and pH was adjusted to 7.0 with NaOH. 100 μL of this was administered intraperitoneally. The prodrug was formulated in a mixture of DMSO, poly(ethylene glycol) (PEG) 400, and phosphate-buffered saline (PBS) (volume ratio 2:19:19). 1 h after the **vitC** injection, 100 μL of the prodrug was administered through IP. 3 mice per group were used. *Escalation Study:* Escalating IP dosages were administered of **vitC** (1 g/kg, 2 g/kg, 3 g/kg, and 4 g/kg) against fixed prodrug doses (5 mg/kg, 10 mg/kg, and 20 mg/kg) until serious adverse events were observed or the maximum dosage was reached (20 mg/kg prodrug in combination with 4 g/kg **vitC**). Dose escalations were conducted with a one-day interval, and weights were documented on the second day. *5-dose Study:* To identify a safe dose of the **vitC** for an *in vivo* efficacy study, decreased doses of **vitC** (500 mg/kg, 750 mg/kg, and 1 g/kg) and the prodrug (5 mg/kg, 10 mg/kg, and 20 mg/kg) (IP injection) were given to the female CD-1 mouse (three mice for each dose) each day until a dose was administered with no signs of weight loss for all mice over a period of 5 d. Once the dosing was completed, animals were observed for another 2 d to observe delayed-onset toxicity effects. Animals with the following signs were euthanized: weight loss of 20% from the initial weight or more, the inability to rise, ambulate, or reach food and water for over 3 d, and the presence of a labored respiration.

### 2.15 In vivo efficacy study with xenograft models

Seven-week-old Immune-deficient female nude mice were anesthetized with isoflurane and injected subcutaneously with cancer cells (MDA-MB-468) suspended in a 1:1 solution of Matrigel (Corning: 354248) and Dulbecco's Modified Eagle Medium (DMEM) media. All cancer cells were obtained from the American Type Culture Collection (ATCC) and were negative for bloodborne pathogens. Cell numbers for each inoculation (100 μL per mouse to the subcutaneous area of the flank) were 5 × 10^6^. Animals were monitored daily for palpable tumors, and animal weights were recorded weekly before/after the compound was administered. When the tumors reached treatment size (200 mm^3^), the mice were randomized to treatment groups (4 groups with 3 mice per group). A vehicle group, **vitC** group, prodrug group, and the combination group. Each was given IP doses each day (5 d per week) for 10 weeks. For the combination group, **vitC** IP injection was given 1 h prior to the prodrug IP injection. **vitC** was dissolved in DI water and pH adjusted to 7.0 with NaOH. The prodrug was formulated in a mixture of DMSO, poly(ethylene glycol) (PEG) 400, and phosphate-buffered saline (PBS) (volume ratio 2:19:19). The volume of injection for both compounds was 100 μL at a concentration of 3.0 mg/kg of the prodrug and 500 mg/kg of **vitC**. Mice were weekly weighed, and tumor sizes were measured using electronic calipers every 7 d. The tumor volume was calculated as follows:

$$V=\left(L\;x\;W^{2}\right)/2$$

At the end of the study period, all tumors, hearts, lungs, livers, kidneys, brains, and spleen were harvested, weighed, and stored in −80 °C for further analysis.

### 2.16 Hematoxylin and eosin (H&E) staining

For histological morphometry and apoptosis analysis, tumor, and major organs, including heart, liver, kidney, lung, spleen, and brain were fixed with 10% formalin, later embedded in paraffin and cut into 5-μm-thick sections and stained with hematoxylin and eosin. Stained slides were Imaged using Hamamatsu WSI imager and analyzed using NDP.view2 software.

### 2.17 In vivo optical imaging

*Metabolic Activity:* To assess tumor metabolic activity, one mouse from each treatment group (Vehicle, **vitC**, **FAN-NM-CH_3_**, and Combination) was selected at week 8 of the treatment. A 100 nmol IRDye 800CW 2-DG Optical Probe (LI-COR: 926-08946), with excitation/emission wavelengths of 770 nm/790 nm, was reconstituted in 2 mL of sterile 1X PBS to achieve a final concentration of 0.05 nmol/μL 100 μL (5 nmol) of this stock solution was administered intravenously via the tail vein. 24 h after injection, the mice were euthanized and imaged for tumor metabolic activity using an *in vivo* imaging instrument. The probe signal was quantified using ImageJ software. Note that the probe is processed through the liver, the major site of glycolysis, and excreted through the kidneys and bladder, causing increased background when imaging these regions. *ROS Detection:* To investigate ROS levels at the tumor site, 4 mice with similar tumor size were left untreated for five weeks after the implantation of cancer cells, so the tumor can grow to a suitable size. During week 6 and 7, these four mice received similar treatment as the efficacy study by IP injection of Vehicle, **vitC**, **FAN-NM-CH_3_**, and Combination. At the end of week 7, 1 mg of the ROS Brite 700 probe (ATT Bioquest: 16004), with excitation/emission wavelengths of 680 nm/700 nm, was reconstituted in 2 mL of sterile 1X PBS to achieve a final concentration of 0.5 μg/μL 100 μL (50 μg) of this stock was administered intratumorally to each mouse under anesthesia with isoflurane 1 h after their daily treatments. Mice were euthanized after 4 h post-probe administration and imaged using an Odyssey Sa imager to determine relative ROS levels in different groups. The ROS signal was quantified using ImageJ software.

## 3 Results

### 3.1 The combination of **vitC** and **FAN-NM-CH_3_** results in potent and selective killing of cancer cells *in vitro*

**Fig. 2:**
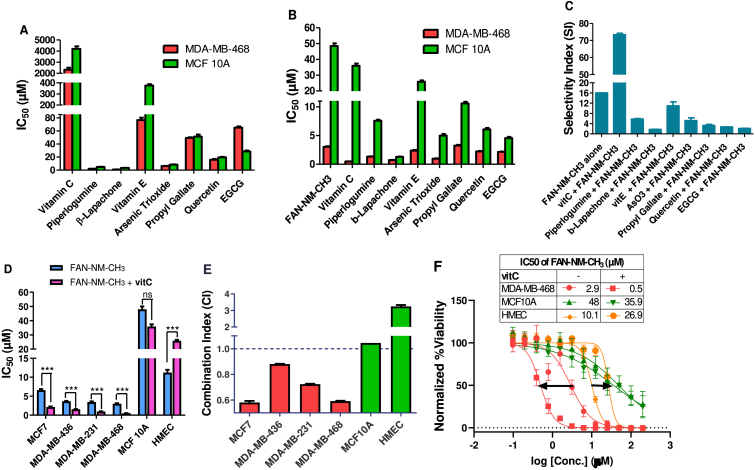
**The cytotoxicity and selectivity of various prooxidants, FAN-NM-CH_3_ alone or combination therapy on various cancer and normal cells** (**A**-**C**: data obtained with MDA-MB-468 cancer cells and MCF10A normal cells; D-F: data obtained with various cancer and normal cell lines). (**A**) The IC_50_ values of prooxidants alone; (**B**) The IC_50_ values of **FAN-NM-CH_3_** alone or in combination with MAXSD of prooxidants; (**C**) Selectivity Index (SI) of **FAN-NM-CH_3_** alone or in combination with prooxidants; (**D**) The effect of MAXSD **vitC** on IC_50_ values of **FAN-NM-CH_3_** in various tumor and normal cell lines; (**E**) Combination Index of combination therapy for different tumor and normal cell lines; (**F**) A representative example of IC_50_ curve of **FAN-NM-CH_3_** with or without **vitC** in cancer (MDA-MB-468) and normal (MCF10A and HMEC) cells. [the data represent three independent experiments performed in triplicate (n = 3)].

To evaluate whether prooxidants can selectively enhance the activity of ROS-responsive prodrugs, we assessed the cytotoxicity of multiple prooxidants alone and in combination with the ROS-responsive prodrug **FAN-NM-CH_3_** in cancer and normal cells (Fig. 1 and Table S1). Triple-negative breast cancer (TNBC) MDA-MB-468 cells and non-tumorigenic MCF10A mammary epithelial cells were selected for initial studies (Fig. 2A–C). Among the prooxidants tested, including piperlongumine, β-lapachone, vitamin E, arsenic trioxide, propyl gallate, quercetin, and EGCG [7] **vitC** demonstrated the most favorable safety and selectivity profile, as indicated by its high IC_50_ values in both cancer and normal cells (Fig. 2A, fig. S1, A to E, and Tables S1 and S2). At its maximum safe dose (MAXSD), **vitC** significantly enhanced the anticancer activity and selectivity of **FAN-NM-CH_3_**, achieving a selectivity index (SI; defined as the ratio of IC_50_ values in normal cells to those in cancer cells) of 73 (Fig. 2B and C). In MDA-MB-468 cells, 1 mM **vitC** lowered the IC_50_ of **FAN-NM-CH_3_** from 3 μM to 0.5 μM, while producing only a modest reduction in MCF10A cells (48 μM to 36 μM) (Fig. 2, B and F). Similar trends were observed with other cancer and normal cell lines (Fig. 2D and fig. S2, A to D). These data suggest that **vitC** and ROS-responsive prodrug **FAN-NM-CH_3_** act synergistically in cancer cells but not in normal cells, which was confirmed by combination index (CI) analysis using the Chou and Talalay method (synergism: CI < 1.0) (Fig. 2D) [29]. Strong synergy was observed across various cancer cell lines (CI = 0.58–0.87) while normal cells exhibited additive or antagonistic interactions (Fig. 2D and E, and Table S3). A key finding was the differential response in normal cell lines. While MCF10A cells showed only a marginal change in prodrug sensitivity (IC_50_: 48 μM alone vs. 35.9 μM with **vitC**), primary HMECs exhibited a clear antagonistic effect (CI = 3.2), with the IC_50_ of FAN-NM-CH_3_ increasing from 10.1 μM alone to 26.9 μM in combination with **vitC**. This protective effect in HMEC, which contrasts with the synergistic killing in cancer cells, significantly widens the therapeutic window and highlights a major advantage of this strategy: **vitC** selectively potentiates tumor cytotoxicity while potentially safeguarding certain normal tissues. Collectively, these findings identify **vitC** as an optimal prooxidant partner for ROS-responsive prodrugs and establish the **vitC** + **FAN-NM-CH_3_** combination as a powerful and selective strategy for targeted tumor killing.

**Fig. 3:**
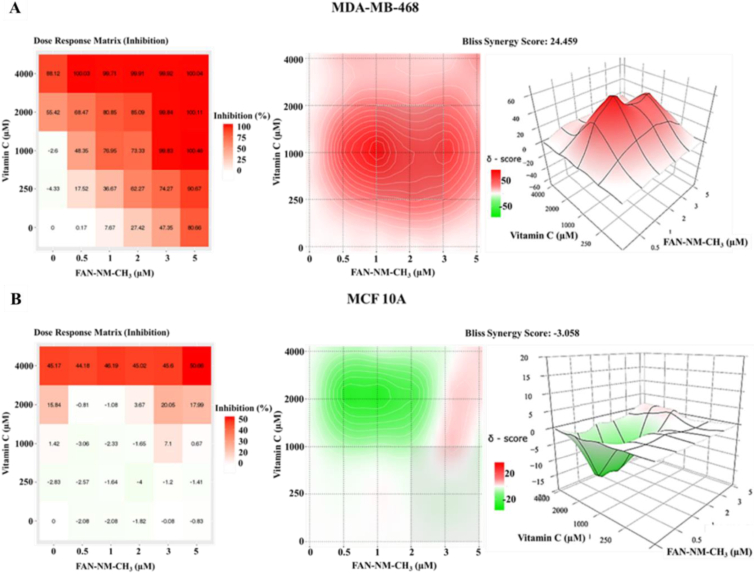
**The drug synergy test for vitC and FAN-NM-CH_3_ by SynergyFinder 3.0 with Dose Response Matrix (left), the drug interaction 2D (middle) and 3D (right) landscapes based on the Bliss mode**l. The synergy score is shown by Red (>0) and Green (<0). A synergy score of < −10 means the interaction is likely to be antagonistic, between −10 and 10 means it's likely to be additive, and >10 means it is likely to be synergistic. Tests were performed on data generated from cytotoxicity studies on (**A**) MDA-MB-468 and (**B**) MCF10A cells when treated for 48 h and data was normalized with their respective untreated controls. All cytotoxicity data used for the synergy score represents 3 independent replicate experiments, mean ± SD (n = 3).

To further validate these findings under multidimensional dose conditions and minimize false-positive synergy, combination studies were analyzed using SynergyFinder 3.0 [31]. A 5 x 5 concentration matrix was designed (0-5 μM **FAN-NM-CH_3_** and 0-4.0 mM **vitC**) (Fig. 3 and Fig. S3). Cell viability data analyzed using SynergyFinder 3.0 (Bliss Independence model) revealed high synergy scores (17.078–24.459) in cancer cells, with large synergy regions (red) evident on both 2D and 3D Bliss independence landscapes (Fig. 3A, and Fig. S3). In contrast, normal cells (MCF10A) displayed a large antagonistic region (green) (Fig. 3B), confirming that **vitC** selectively sensitizes cancer cells to **FAN-NM-CH_3_** while sparing normal cells.

### 3.2 The synergy between **vitC** and **FAN-NM-CH_3_** is driven by **vitC**-induced H_2_O_2,_ redox-active iron, and limited catalase activity in cancer cells

**Fig. 4:**
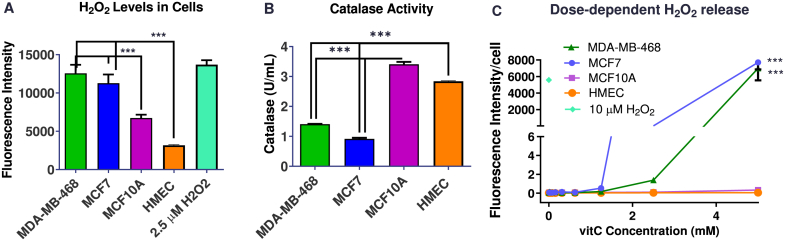
**H_2_O_2_ level in different cell lines and catalase activity.** (**A**) Extracellular H_2_O_2_ levels in various cell lines (n = 3) (H_2_O_2_ level was measured by Amplex Red in culture medium immediately after removing the cells); (**B**) Catalase activity in different cell lines (n = 3); (**C**) Dose-dependent H_2_O_2_ generation induced by **vitC**. The significance was determined by one-way ANOVA followed by Dunnett to compare all columns (n = 4), (*) P < 0.05, (***) p < 0.0001 vs normal cell MCF10A and HMEC.

**H_2_O_2_ generation and catalase activity shape selective activation of FAN-NM-CH_3_.** To elucidate the mechanism underlying the synergistic anticancer effects of **vitC** and **FAN-NM-CH_3_**, we first asked whether the synergy requires H_2_O_2_-mediated activation of the boronic acid moiety. Chlorambucil (**Chlor**), a DNA alkylating agent lacking an H_2_O_2_-responsive moiety [32] was used as a control (fig. S2, C to F). In contrast to **FAN-NM-CH_3_**, the combination of **Chlor** and **vitC** showed an antagonistic effect (CI = 1.38, DRI = 1) (Fig. S2F), demonstrating that the H_2_O_2_-responsive boronic acid functionality in **FAN-NM-CH_3_** is essential for synergy. Because **FAN-NM-CH_3_** activation is H_2_O_2_-dependent, we next quantified basal and **vitC**-induced H_2_O_2_ levels together with catalase activity—a key enzyme that breaks down H_2_O_2_ to water and oxygen. MDA-MB-468, MCF7, MCF10A and HMEC cells were selected as representative models for TNBC, HR + breast cancer, and non-tumorigenic breast epithelial cells, respectively. Cancer cells displayed elevated basal H_2_O_2_ levels (Fig. 4A) and markedly reduced catalase activity relative to normal cells (Fig. 4B and Fig. S4). **VitC** selectively increased H_2_O_2_ in cancer cells (Fig. 4C, Fig. S6–S7), while producing minimal changes in normal cells, indicating that **vitC**-induced H_2_O_2_ accumulation is a key determinant of selective **FAN-NM-CH_3_** activation. Consistent with this model, normal cells with high intrinsic catalase activity and low H_2_O_2_ burden effectively prevented H_2_O_2_ accumulation and remained resistant to the **vitC** + **FAN-NM-CH_3_** combination. HMECs, which had the lowest basal H_2_O_2_, showed the strongest resistance and even antagonism (Fig. 1 E and F and Fig. 4A).

**Fig. 5:**
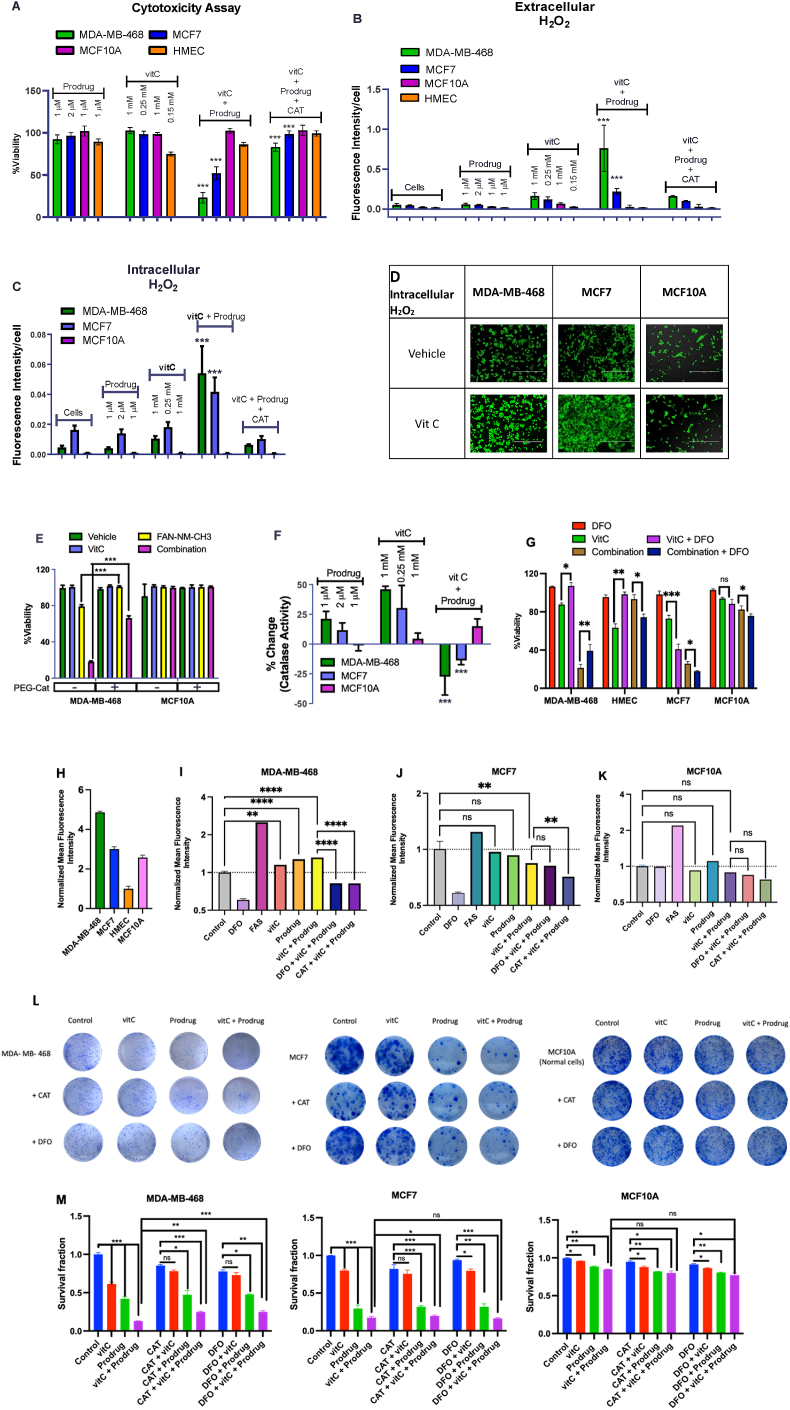
Correlation between cell viability and H_2_O_2_ level as well as catalase activity. (**A**) Cell viability upon treatment under different conditions; (**B**) Extracellular H_2_O_2_ levels measured by Amplex Red Hydrogen Peroxide/Peroxidase Assay (Invitrogen: A22188); (**C**) Intracellular H_2_O_2_ levels assessed by Hydrogen Peroxide Assay Kit (abcam: ab138874) that uses cell-permeable AbGreen indicator to quantify H_2_O_2_ in live cells; (**D**) Representative images of intracellular H_2_O_2_ levels measured by Hydrogen Peroxide Assay Kit (abcam: ab138874) (Scale bar = 400 μm); (**E**) PEG-catalase-mediated intracellular H_2_O_2_ quenching in MDA-MB-468 and MCF10A cells; (**F**) The change of catalase activity in different cell lines treated under different conditions (n = 3); **(G)** Effect of iron chelation by 100 μM deferoxamine (DFO) on **vitC** mediated cytotoxicity and combination treatment; (**H**) Basal intracellular labile Fe^2+^ levels in MDA-MB-468, MCF7, MCF10A, and HMEC cells measured using BioTracker Far-Red flow cytometry; (**I–K**) Treatment induced changes in the intracellular labile Fe^2+^ pool in MDA-MB-468, MCF7, and MCF10A cells following the indicated treatments; (**L-M**) Long-term clonogenic survival assays and normalized survival fractions of MDA-MB-468, MCF-7, and MCF-10A cells following the indicated treatments with DFO and CAT. MDA-MB-468, MCF7, MCF10A, and HMEC cells were treated with MAXSD of **FAN-NM-CH_3_** (1 μM, 2 μM, 1 μM and 1 μM), or **vitC** (1 mM, 0.25 mM, 1 mM and 0.15 mM), or combination of **FAN-NM-CH_3_** and **vitC** at 37 °C for 48 h. The significance was determined by one-way ANOVA followed by Dunnett to compare all columns (n = 4), (*) P < 0.05, (***) p < 0.0001 vs. MCF10A (Due to the limited proliferative capacity of primary HMECs under extended culture, sufficient cell numbers could not be obtained for multi-condition iron modulation assays; therefore, only basal labile Fe^2+^ levels were quantified in HMECs).

To directly probe the functional role of H_2_O_2_, cells were treated with **vitC**, **FAN-NM-CH_3_**, or their combination in the presence or absence of catalase (Fig. 5, Fig. S8–S11, and Table S4). The combination of safe doses of **vitC** and **FAN-NM-CH_3_** lead to significant cancer cell death (∼40%-80%), while catalase addition markedly diminished this cytotoxicity by quenching H_2_O_2_ and preventing **FAN-NM-CH_3_** activation (Fig. 5A and B, and Fig. S8 and S9). These effects persisted across multiple dose ranges (Fig. S12–S16). Combination treatment generated pronounced extracellular and intracellular H_2_O_2_ accumulation selectively in cancer cells (up to ∼5-fold in MDA-MB-468), closely paralleling the degree of cytotoxicity observed (Fig. 5B–D). FAN-NM-CH_3_ alone did not elevate H_2_O_2_, confirming that **vitC** is the initiating oxidative driver. MDA-MB-468 cells, which accumulated the highest H_2_O_2_ levels, also exhibited the strongest synergy. These findings underscore the essential role of **vitC**-induced H_2_O_2_ in the activation of **FAN-NM-CH_3_** and the resultant cytotoxic effects on cancer cells.

Because extracellular catalase is not membrane-permeable, these experiments primarily reflect the effects of extracellular H_2_O_2_. To test whether intracellular H_2_O_2_ contributes to prodrug activation, we used polyethylene glycol–conjugated catalase (PEG-CAT), a cell-permeable catalase mimetic, which enables intracellular scavenging of H_2_O_2_ without genetic perturbation of cellular redox systems. PEG-CAT partially rescued viability in MDA-MB-468 cells treated with the **vitC** + **FAN-NM-CH_3_** combination (Fig. 5E, Fig. S5), supporting a role for intracellular H_2_O_2_ in prodrug activation. The more modest rescue relative to native catalase likely reflects limited intracellular accumulation or catalytic efficiency. Together, these data indicate that both extracellular and intracellular H_2_O_2_ contribute to **FAN-NM-CH_3_** activation.

Interestingly, **vitC** or **FAN-NM-CH_3_** alone induced modest upregulation of catalase in cancer cells, consistent with an adaptive response to moderate oxidative stress (10%–20% increase for **FAN-NM-CH_3_**; 26%–46% for **vitC**) (Fig. 5F). In contrast, combination treatment suppressed catalase activity (14–27%), resulting in sustained H_2_O_2_ accumulation (2–7-fold increases) (Fig. 5B–D). Normal cells maintained high catalase activity under all treatment conditions (Figs. 4B and 5F), effectively preventing H_2_O_2_ buildup and conferring resistance to the combination therapy. These findings explain how **vitC** and **FAN-NM-CH_3_** cooperatively overwhelm antioxidant defenses in cancer cells but not in normal epithelial cells.

**Convergence of H_2_O_2_ flux and labile iron availability governs tumor-selective cytotoxicity.** Pharmacologic ascorbate has been shown to increase intracellular labile iron and potentiate H_2_O_2_-dependent toxicity in multiple cancer models [[33], [34], [35], [36], [37]] and aggressive breast cancer cells often exhibit dysregulated iron homeostasis due to reduced ferroportin expression and altered hepcidin regulation [38]. Given that pharmacologic ascorbate generates extracellular H_2_O_2_ capable of diffusing intracellularly [9,10] and engaging in iron-catalyzed redox cycling, we hypothesized that the magnitude of cytotoxicity downstream of prodrug activation could be influenced by the availability of intracellular redox-active iron within the labile iron pool (LIP). To test this hypothesis, we determined the effect of an iron chelator deferoxamine (DFO) on cell viability and its correlation with the level of intracellular LIP. Iron chelation by DFO produced cell-type–dependent rescue effects that closely tracked with intracellular Fe^2+^ levels and LIP responsiveness (Fig. 5 G-K).

In MDA-MB-468 TNBC cells, DFO increased viability following **vitC** + **FAN-NM-CH_3_** treatment (Fig. 5G), indicating that redox-active iron contributes to maximal cytotoxicity. These cells exhibited high basal Fe^2+^ levels that were maintained or enhanced following **vitC**, prodrug, or combination treatment (Fig. 5H and I), creating a permissive intracellular environment for iron-catalyzed oxidative amplification. Both DFO and catalase reduced Fe^2+^ signal intensity and partially rescued viability, supporting a model in which H_2_O_2_-dependent prodrug activation is coupled to robust Fe^2+^-mediated redox cycling that sustains secondary radical generation and exacerbates oxidative damage.

In contrast, ER-positive MCF-7 cells exhibited a more constrained labile iron pool, characterized by lower basal Fe^2+^ levels and limited inducibility (Fig. 5, H and J) [38]. **VitC** and combination treatment slightly reduced Fe^2+^ levels, and catalase markedly diminished the detectable Fe^2+^ signal (Fig. 5J), indicating that the measurable LIP in these cells is coupled to ongoing H_2_O_2_ flux. Despite effective iron depletion by DFO, chelation failed to rescue viability and instead modestly reduced cell survival (Fig. 5G), consistent with iron deprivation–induced metabolic stress rather than protection from oxidative damage. These findings indicate that in MCF-7 cells, H_2_O_2_-dependent prodrug activation is sufficient to induce cytotoxicity, whereas iron-mediated oxidative amplification is not a dominant determinant of outcome.

Importantly, this iron-dependent modulation was not observed in normal mammary epithelial models. MCF10A and primary HMECs exhibited low basal Fe^2+^ levels (Fig. 5H) and a narrow dynamic range of LIP modulation across all treatment conditions (Fig. 5K). In these cells, **vitC** and combination treatment further reduced intracellular Fe^2+^ levels, and catalase produced a similar modest decrease (Fig. 5K), indicating minimal engagement of H_2_O_2_-driven iron redox cycling. Consistent with this constrained iron biology, neither catalase nor DFO provided a protective effect against cytotoxicity. While catalase did not significantly alter cell viability, DFO modestly reduced survival, reflecting iron deprivation–induced metabolic stress rather than rescue from oxidative damage [39]. Accordingly, MCF-10A cells, which are intrinsically resistant to the **vitC** + **FAN-NM-CH_3_** combination, showed no significant change in viability upon DFO pretreatment (Fig. 5G). Similarly, HMECs did not benefit from iron chelation; instead, DFO modestly reduced viability (Fig. 5G), consistent with the known sensitivity of non-transformed epithelial cells to iron deprivation [39].

Together, these data support a two-tiered mechanism of tumor-selective cytotoxicity [1]: H_2_O_2_-dependent prodrug activation, which is required across all cell types, and [2] Fe^2+^-dependent oxidative amplification, which determines the magnitude and durability of cell killing. Within this framework, partial rescue by catalase or DFO reflects disruption of upstream redox inputs after irreversible downstream damage has already occurred, consistent with a temporal hierarchy of oxidative initiation followed by execution-level DNA damage. Collectively, **vitC**-mediated activation of **FAN-NM-CH_3_** exploits a cancer-specific oxidative environment defined by elevated H_2_O_2_ flux, limited antioxidant buffering, and increased availability of redox-active iron, while normal epithelial cells constrain both H_2_O_2_ propagation and iron-catalyzed redox cycling, thereby conferring resistance to the combination therapy.

**Clonogenic validation of durable redox-dependent cytotoxicity.** While short-term metabolic viability assays (CellTiter-Glo) capture acute cytotoxic responses, redox-mediated DNA damage, particularly when amplified by iron-dependent oxidative cascades, may manifest as delayed or irreversible loss of proliferative capacity. To rigorously assess whether the mechanistic differences observed above translate into durable cell fate outcomes, we therefore performed long-term clonogenic survival assays, the gold-standard *in vitro* endpoint most predictive of *in vivo* tumor response. This approach is especially relevant for pharmacologic ascorbate, whose cytotoxicity is highly dependent on cell density, redox context, and post-treatment recovery.

Clonogenic analysis revealed near-complete ablation of colony formation in MDA-MB-468 cells following **vitC** + **FAN-NM-CH_3_** treatment, with partial rescue by catalase or DFO (Fig. 5L and M), confirming that both H_2_O_2_ flux and redox-active iron contribute to durable proliferative failure. In contrast, MCF-7 cells exhibited profound clonogenic suppression that was not rescued by iron chelation, reinforcing the conclusion that iron-mediated amplification is not the dominant determinant of outcome in this model. Normal epithelial cells (MCF10A and HMECs) retained substantially greater clonogenic capacity under identical treatment conditions, consistent with their limited iron availability and preserved antioxidant buffering.

Together, these results validate and extend the acute viability findings, demonstrating that tumor-selective cytotoxicity arises from the convergence of H_2_O_2_-dependent prodrug activation and differential intracellular iron biology, which governs not only the extent but also the durability of oxidative damage.

### 3.3 The combination of **vitC** and **FAN-NM-CH_3_** exacerbates DNA damage and cell apoptosis in cancer cells

**Fig. 6:**
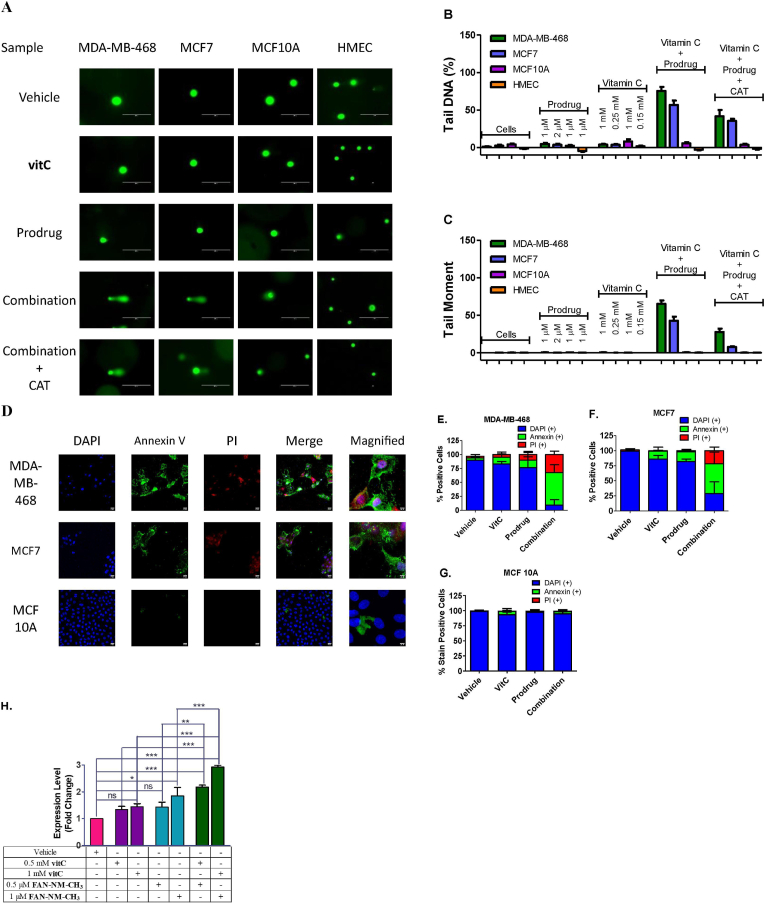
**A combination of safe doses of vitC and FAN-NM-CH_3_ leads to DNA damage and p53 upregulation, triggering cancer cell apoptosis**. (**A-C**) The alkaline comet assay analysis of MDA-MB-468, MCF7, MCF10A, and HMEC cells after treatment with vehicle, **vitC**, **FAN-NM-CH_3_**, and the combination: (**A**) Comet Images taken with EVOS Digital Inverted Microscope at 20X magnification; (**B**) Quantification of % DNA in the tail of the comets; (**C**) Quantification of Tail moment (Images were analyzed by TriTek CometScore Software). Each data point represents 3 independent replicate experiments, and the data are presented as the mean ± SD (n = 3). (**D-G**) Determination of cell death pathway by Annexin V/PI/DAPI staining using fluorescence confocal microscopy: (**D**) The representative images of Annexin V/PI/DAPI staining of cells treated with combination (The scale bar represents 10 μm for the overall image and 2.5 μm for the magnified section); (**E-G**) quantification of stain positive cell population (%) using software ImageJ, n = 3. Images represent at least 5 fields observed in 3 different preparations after 48 h of treatment. **(H)** p53 mRNA expression level (n = 3) of MDA-MB-468 cells treated with **vitC**, **FAN-NM-CH_3_**, or combination. Data was normalized with relative GAPDH mRNA levels and fold change calculated with 2^−ΔΔCt^ method of Lovak and Schmittgen. The significance was determined by one-way ANOVA followed by Dunnett to compare all columns (n = 7), (*) P < 0.05, (***) p < 0.0001 vs vehicle.

We hypothesize that **vitC**-induced H_2_O_2_ accumulation activates the prodrugs, releasing alkylating intermediates that induce DNA damage, ultimately driving cell death. Because the highly reactive nitrogen mustard intermediates generated from ROS-responsive prodrugs are extremely short-lived and not amenable to direct chemical quantification in biological systems, downstream cellular DNA damage serves as a functional surrogate for effective prodrug activation. Accordingly, cellular DNA damage was evaluated using an alkaline comet assay in MDA-MB-468, MCF7, MCF10A, and HMEC cells. DNA damage, indicated by longer comet tails, was observed in cancer cells treated with **vitC** + **FAN-NM-CH_3_** combination, whereas minimal damage was detected with either agent alone or in normal cells (Fig. 6A). Quantification of the comet tail, tail moment, and tail olive moment by TriTek CometScore Software revealed the extent of DNA damage, in the order of MDA-MB-468 (∼75%) > MCF7 (∼57%) > MCF10A (∼5%) > HMEC (∼1%) (Fig. 6B and C, Fig. S17, and Tables S5 and S6). This trend closely aligned with the synergistic cytotoxicity observed for the combination therapy.

To assess whether this DNA damage is mediated by **vitC**-derived H_2_O_2_, catalase was co-administered to scavenge extracellular H_2_O_2_ prior to combination treatment. Catalase markedly attenuated combination-induced DNA damage, as evidenced by reduced comet tail lengths (Fig. 6A, catalase rescue panel) and corresponding decreases in quantitative metrics (Fig. 6B and C). Specifically, catalase reduced tail DNA% from 75% to 42% in MDA-MB-468 cells, from 57% to 36% in MCF7 cells, and from 5.5% to 3.5% in MCF10A cells (Fig. 6B). Similarly, catalase reduced tail moment values from 65 to 28 (MDA-MB-468), from 43 to 8 (MCF7), and from 0.62 to 0.28 (MCF10A). No significant effect was observed in HMECs, as no detectable DNA damage was induced under any treatment conditions (Fig. 6C). These findings indicate that catalase partially rescues combination-induced DNA damage by intercepting **vitC**-generated H_2_O_2_. Consistent with this interpretation, AbGreen fluorescence imaging demonstrated complete quenching of **vitC**-induced H_2_O_2_ signals upon catalase addition (Table S4), supporting a requirement for H_2_O_2_ in **FAN-NM-CH_3_** activation and downstream DNA damage.

Because DNA damage can engage apoptotic signaling pathways when not efficiently repaired, we next examined whether the **vitC/FAN-NM-CH_3_** combination promoted apoptosis. Annexin V/PI/DAPI staining revealed a significant increase in apoptotic cell populations following combination treatment, particularly in breast cancer cells, while normal epithelial cells remained largely protected (Fig. 6D–G). DNA alkylating agents, such as chlorambucil, are reported to induce cell apoptosis via upregulation of the p53 gene, a key regulator of apoptosis [32]. To understand the combination therapy's effect on p53 expression, we quantified p53 mRNA levels in MDA-MB-468 cells. Low-dose **vitC** or **FAN-NM-CH_3_** slightly increased p53 expression (∼0.3-fold), while higher **FAN-NM-CH_3_** doses induced a ∼0.9-fold increase. Combination of safe doses of **vitC** (0.5 mM-1.0 mM) and **FAN-NM-CH_3_** (0.5-1.0 μM) resulted in significant p53 upregulation (1.2- to 1.9-fold, depending on doses) (Fig. 6H). These results suggest that slightly increased H_2_O_2_ levels induced by **vitC** alone has a minimum effect on p53 expression, whereas H_2_O_2_-mediated **FAN-NM-CH_3_** activation drives increased DNA damage and p53 upregulation, leading to cancer cell apoptosis.

### 3.4 The combination of **vitC** and **FAN-NM-CH_3_** leads to regression of established tumors in mice without adverse effects

The efficacy and selectivity of the combination of **vitC** and **FAN-NM-CH_3_** was further evaluated *in vivo* with xenograft mouse model. Initially, we used CD-1 mice to determine the safe doses of combination therapy (Fig. S18–S21 and Table S9). No apparent signs of toxicity were observed with a combination of 5.0 mg/kg or 10 mg/kg of **FAN-NM-CH_3_** with **vitC** at doses of 0.5 - 3.0 g (Fig. S18C–S18E). Mice treated with combination showed weight gains comparable to controls. Monitoring mouse health and welfare with a Mouse Intervention Scoring System (MISS) adapted from Koch et al. and Paster et al., [40,41] suggested that all mice treated with combination showed normal behavior with a terminal score >10 (fig. S18, G and H, and SI Table S8). However, 20 mg/kg dose of **FAN-NM-CH_3_** exhibited significant weight loss when combined with **vitC** at doses of 500 - 1000 mg/kg (Fig. S18F). Therefore, for the *in vivo* efficacy study, a low-dose combination of **FAN-NM-CH_3_** (3 mg/kg) and **vitC** (500 mg/kg) was selected to evaluate synergistic effects while minimizing the influence of single-agent toxicity.

**Fig. 7:**
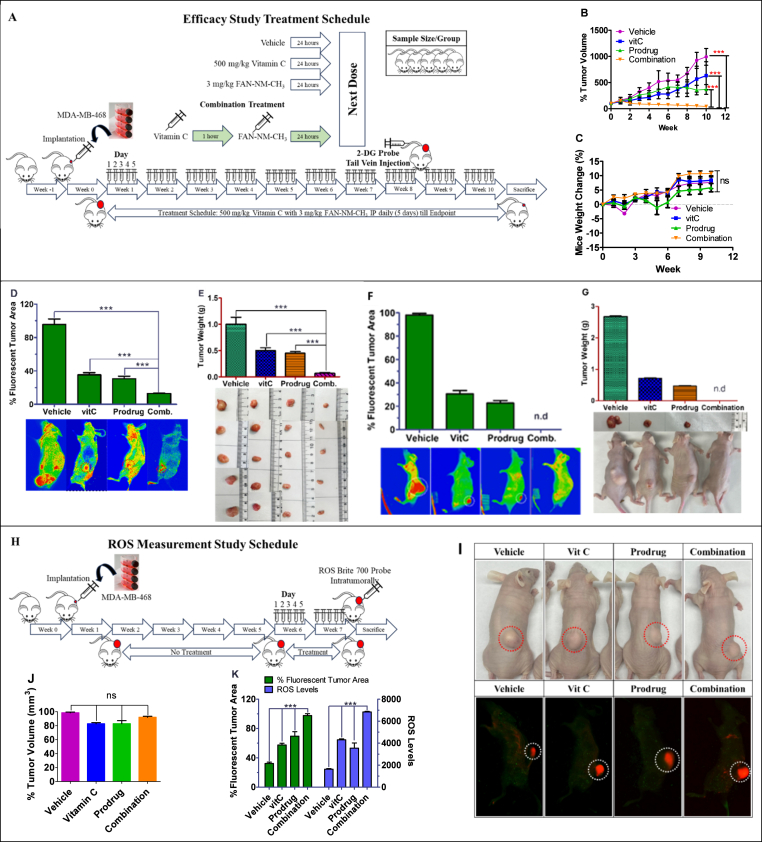
In vivo antitumor efficacy and safety. (A) Scheme of the treatment timeline for the *in vivo* efficacy study and 2DG probe administration; (B) Tumor volumes measured by caliper; (C) Time-dependent mice weight change %; (D) Tumor regression indicated by the whole mice image and quantification of the tumor fluorescent area using ImageJ software; (E) Tumor weights at the end of 10-week treatment and photos of the harvested tumors; (F and G) Tumor sizes after 23 weeks including10-week treatment and 13-week post-treatment observation: (F) the whole mice imaging and quantification of the tumor fluorescent area using ImageJ software (n.d.: not detected); (G) tumor weight, appearance, and photos of the harvested tumors; (H–K): Evaluation of ROS level in mouse tumor: (H) ROS measurement study schedule (mice received ROS Brite 700 probe intratumorally five weeks after inoculation with MDA-MB-468 cells); (I) Mice appearance and fluorescent images; (J) Tumor volumes measured by caliper; (K) The fluorescent tumor area (%) and weighted fluorescent index. Fluorescent tumor area (%) and mean fluorescence intensity were quantified by ImageJ software. Weighted Fluorescence Index is defined as the product of % Fluorescent Tumor Area and the corresponding Mean Fluorescence Intensity. Each data point represents 3 independent replicate experiments, and the data are presented as the mean ± SD (n = 3). The significance was determined by one-way ANOVA followed by Dunnett to compare all columns (n = 4), (*) P < 0.05, (***) p < 0.0001 vs combination-treated mice.

Athymic nude mice xenografted with human tumor cell lines were used to evaluate the efficacy and selectivity of the combination treatment (Fig. 7). MDA-MB-468 breast cancer cells were implanted subcutaneously in nude mice, resulting in tumor formation within one week (Fig. 7A). Mice were divided into four groups (n = 5/group): vehicle, **vitC** (500 mg/kg), **FAN-NM-CH_3_** (3 mg/kg), and a combination of **vitC** (500 mg/kg) and **FAN-NM-CH_3_** (3 mg/kg). Treatments were administered IP for five days per week over ten weeks (Fig. 7 and Fig. S22–S26). For the combination group, **vitC** was injected 1 h before **FAN-NM-CH_3_** administration (Fig. 7A). Tumor size, body weight, health, and behavior were monitored weekly. Tumor volumes were measured weekly using calipers and the whole mice imaging conducted at week 8. The combination treatment not only inhibited tumor growth but also induced significant tumor shrinkage (Fig. 7B, and Fig. S22). In comparison with **vitC** or **FAN-NM-CH_3_** alone, the combination of the two greatly enhanced their *in vivo* efficacy without causing side effects (Fig. 7B and C, and Fig. S23). Tumors in combination-treated mice decreased to approximately 80% of their initial size after three weeks and 40% after ten weeks (Fig. 7B). In contrast, vehicle-treated mice experienced a 1000% increase in tumor size. While **vitC** or **FAN-NM-CH_3_** alone slightly inhibited tumor growth, neither caused tumor shrinkage (Fig. 7B). By week 10, the average tumor volume in the combination group (42 ± 7.5 mm^3^) was only 4% of the control group (991 ± 226.4 mm^3^), compared to 64% and 37% for **vitC** (631 ± 458.9 mm^3^) and **FAN-NM-CH_3_** (367 ± 185.4 mm^3^), respectively. The tumor growth inhibition rate [IR (%) = [1 − (mean volume of treated tumors)/(mean volume of control tumors)] × 100] was 96% for the combination, 63% for FAN-NM-CH_3_ alone, and 36% for **vitC** alone.

Whole mouse fluorescence imaging confirmed significant tumor regression in the combination group (Fig. 7D). At week-8, mice were injected with infrared dye 800-conjugated 2-deoxy-d-glucose (2-DG) via tail-vein and imaged in a LI-COR Odyssey infrared scanner (Fig. 7A). Tumors, due to heightened metabolic activity, produced a stronger fluorescent signal than the rest of the body. The combination-treated mouse showed the smallest fluorescent tumor area (13% relative to the vehicle mouse). Tumors excised at week 11 further confirmed these findings. Average tumor weights were significantly lower in the combination group (67.5 ± 26.8 mg) compared to **vitC** (500.0 ± 91.6 mg), **FAN-NM-CH_3_** (450.0 ± 52.4 mg), and vehicle groups (1003.3 ± 185.2 mg) (Fig. 7E). These results demonstrate that the combination of **vitC** and **FAN-NM-CH_3_** leads to regression of established tumors in mice.

To evaluate long-term effects, in a separate experiment, mice were monitored for 13 weeks after the 10-week treatment. Combination-treated mice exhibited no delayed adverse effects or tumor recurrence, with complete tumor regression observed (Fig. 7F and G, and figs. S24, A to D). In contrast, tumors in vehicle, **vitC**, and **FAN-NM-CH_3_** groups grew significantly during this period, with control tumors reaching 2300% of their original size, 800% for **vitC** group, and 600% for **FAN-NM-CH_3_** group (fig. S24, C and D). Whole mice imaging with a 2-DG probe further confirmed our observations (Fig. 7F). No detectable fluorescence (indicating a tumorous area) was found in combination-treated mice, while tumor regions with strong fluorescent signals were detected in control mice, **vitC**-treated mice, and **prodrug**-treated mice. Tumor weights were 2.68 g for control mouse, 0.71 g for **vitC**-treated mouse, and 0.47 g for prodrug-treated mouse (Fig. 7G).

The four groups of nude mice from the *in vivo* efficacy study were monitored for symptoms of toxicity, including body weight changes, appetite loss, lethargy, treatment-related mortality, and organ health (Fig. 7C, figs. S23, S25, S26, and Tables S9 and S10). Mice treated with **vitC** (500 mg/kg), **FAN-NM-CH_3_** (3 mg/kg), or their combination, exhibited no signs of toxicity. All groups gained weight steadily throughout the 10-week treatment period and 13-week post-treatment observation (Fig. 7C and Fig. S23). Weekly scoring confirmed overall terminal scores >10, with no evidence of organ dysfunction (Fig. S24 and S25). Kaplan-Meier analysis, defining an event as tumor volume exceeding 1000% of its original size, demonstrated a significant survival benefit for the combination group relative to all other groups (Fig. S27). Histological analysis of heart, liver, spleen, kidney, lung, and brain tissues from treated mice revealed no significant pathological changes or toxicity (Fig. S26 and Table S11). All organ architecture remained intact, with no inflammation, fibrosis, or cellular damage observed. These findings indicate that the combination treatment is well-tolerated by mice without adverse effects on these major organs. Together, the *in vivo* investigation demonstrated that a combination of **vitC** with ROS-responsive prodrugs leads to potent and selective tumor killing without affecting normal tissues in mice.

Cell culture studies have revealed that ROS levels play a crucial role in the synergistic anticancer efficacy and selectivity of the combination therapy involving **vitC** and **FAN-NM-CH_3_**. To validate this finding *in vivo*, tumor ROS levels were measured using the ROS Brite 700 (RB 700) probe, which fluoresces upon ROS oxidation (Fig. 7H). The study was conducted five weeks after tumor cell inoculation, when all mice had reached a suitable tumor size (Fig. 7I and J). To eliminate potential discrepancies due to tumor volume, the RB 700 probe was injected after two weeks of treatment, when all mice had similar tumor sizes. The tumor volume relative to the vehicle was 92% in combination-treated mice, and 83% in both prodrug and vitC-treated mice (Fig. 7J). In vivo fluorescence imaging on the Odyssey Sa imager indicated that tumor cells were under oxidative stress in all mice (Fig. 7I). The ROS level, as indicated by the % fluorescent tumor area, followed the order: combination-treated mice > **FAN-NM-CH_3_**-treated mice > **vitC**-treated mice > control mice (Fig. 7K). Tumors naturally produce ROS due to their high metabolic activity, but **vitC** and **FAN-NM-CH_3_** further elevated oxidative stress, with the combination treatment showing the highest ROS levels, correlating with the highest antitumor effects. The Weighted Fluorescence Index (fluorescent tumor area × mean intensity) ranked as follows: combination (6833) > vitC (4303) > prodrug (3548) > vehicle (1634) (Fig. 7K). These results strongly suggest a correlation between elevated ROS levels and the enhanced *in vivo* anticancer efficacy of the **vitC** and **FAN-NM-CH_3_** combination therapy.

## 4 Discussion and conclusion

Cancer cells are more susceptible than normal cells to disruptions in redox homeostasis [3,5,42]. Exploiting this vulnerability to selectively target tumors—referred to as oxidative stress–based cancer therapy—has gained increasing attention in recent decades [[2], [3], [4],^6^]. Numerous pro-oxidants have been identified for their ability to induce oxidative stress in cancer cells, showing promising results in certain cases [[2], [3], [4], [5], [6], [7]]. However, challenges such as limited tumor selectivity and insufficient therapeutic durability have hindered their broader clinical application. In this study, we address these limitations by combining pro-oxidants with ROS-responsive prodrugs. This approach leverages pro-oxidants to amplify oxidative stress within tumors, enhancing the sensitivity of cancer cells to ROS-responsive prodrugs and producing synergistic anticancer effects.

Among various prooxidants, **vitC** is an attractive candidate for combination with ROS-responsive prodrugs due to its favorable safety profile in normal tissues. The antitumor potential of **vitC** is well-documented, with extensive research focused on high-dose **vitC** or its combination with standard chemotherapy or radiotherapy to enhance therapeutic outcomes [[43], [44], [45], [46], [47], [48], [49], [50], [51], [52], [53]]. However, clinical results regarding the efficacy of high-dose **vitC**, either as a standalone treatment or in combination with other chemotherapeutic agents, have been inconsistent [[45], [46], [47], [48],^54^,^55^]. For instance, a phase I/II trial in metastatic pancreatic cancer patients reported that high-dose intravenous **vitC** combined with gemcitabine and erlotinib was safe and associated with a positive trend in overall survival compared to historical controls [46,53,56]. In contrast, a phase II trial combining high-dose intravenous **vitC** with docetaxel in patients with metastatic castration-resistant prostate cancer showed no therapeutic benefit and was terminated early, consistent with earlier studies indicating that HDIVC (high-dose intravenous vitamin C) monotherapy is largely ineffective [57]. These inconsistent outcomes highlight the need for strategies to harness **vitC**'s pro-oxidant activity more selectively. In this work, we report a novel application of **vitC** to potentiate the effects of ROS-responsive prodrugs.

Numerous studies have shown that non-toxic prodrugs containing H_2_O_2_-responsive boronate ester or boronic acid groups can be selectively activated by ROS, releasing cytotoxic agents specifically in cancer cells with elevated ROS levels [[14], [15], [16], [17], [18], [19], [20], [21], [22], [23]]. While ROS-activated prodrugs hold promise for improving tumor specificity and minimizing off-target toxicity, challenges remain, including tumor ROS heterogeneity, non-targeted drug distribution, insufficient prodrug activation, and limited therapeutic durability. Our findings demonstrate that **vitC** can amplify tumor oxidative stress and thereby enhance the activation of ROS-responsive prodrugs, providing the first evidence that certain prooxidants can be strategically combined with ROS-responsive prodrugs to overcome these limitations.

**Fig. 8:**
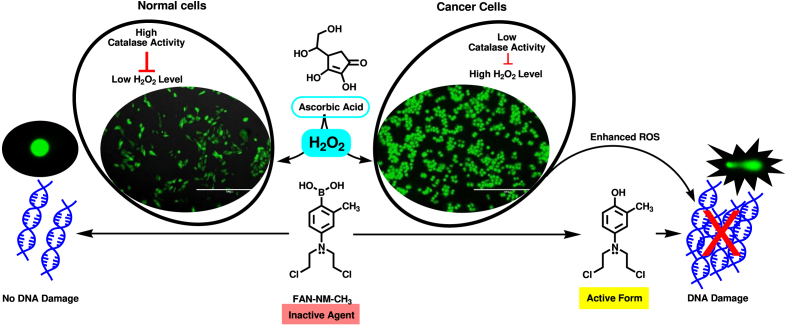
Graphical representation of vitC-induced H_2_O_2_ generation that activates H_2_O_2_-responsive prodrug specifically in cancer cells leading to DNA damage and cell apoptosis.

**VitC** selectively induces H_2_O_2_ generation in cancer cells, triggering activation of ROS-responsive prodrugs and release of cytotoxic species (Fig. 8). Collectively, our results support a redox-mediated activation model in which pharmacologic **vitC** generates extracellular H_2_O_2_ that initiates oxidative deboronation of the prodrug. Diffusible H_2_O_2_ can also enter tumor cells, amplifying intracellular redox reactions and leading to localized formation of highly reactive alkylating intermediates that induce DNA damage and apoptosis. Rather than relying on direct recovery of these transient alkylating intermediates, this study integrates catalase-based functional modulation, cellular H_2_O_2_ measurements, DNA damage assays, and *in vivo* tumor regression—together with prior imaging studies using ROS-responsive theranostic probes as well as isotopically resolved metabolic studies [17,27,28]—to define the site, redox dependence, and biological consequences of prodrug activation. This dual action not only causes DNA damage through prodrug activation but also elevates ROS levels, resulting in extensive cancer cell death via apoptosis.

Tumor selectivity of this combination treatment is primarily governed by differences in catalase activity between cancer and normal cells. Cancer cells, which inherently have lower catalase activity, undergo additional catalase suppression upon treatment with the **vitC**/prodrug combination, leading to substantial H_2_O_2_ accumulation and enhanced prodrug activation. This results in substantial H_2_O_2_ accumulation and enhanced prodrug activation. In contrast, normal cells, characterized by higher basal catalase activity, adapt to treatment-induced oxidative stress by maintaining or upregulating catalase, thereby limiting ROS accumulation and protecting themselves from prodrug activation. In parallel, intracellular labile iron availability governs the magnitude and durability of cytotoxicity downstream of H_2_O_2_-dependent prodrug activation, amplifying oxidative damage through iron-mediated redox reactions.

Furthermore, intracellular redox-active iron amplifies oxidative toxicity through Fenton chemistry, producing highly reactive hydroxyl radicals that exacerbate DNA damage initiated by H_2_O_2_ and prodrug activation. Together, this dual mechanism—prodrug activation by **vitC**-generated H_2_O_2_ and iron-mediated oxidative amplification—likely underlies the strong synergistic killing observed in iron-rich tumors such as triple-negative breast cancer, while sparing normal tissues with lower labile iron pools.

*In vivo* studies using xenograft mouse models further support the relationship between enhanced tumor ROS levels and the synergistic anticancer activity of this combination therapy. Mice treated with a combination of **vitC** and the prodrug showed elevated tumor ROS levels compared with mice treated with either agent alone. Importantly, the combination achieved complete tumor regression without recurrence, significantly outperforming single-agent treatments. Moreover, potent antitumor activity was achieved at relatively low doses while minimizing systemic toxicity, highlighting its potential for treating aggressive cancers such as triple-negative breast cancer.

Although we focused on **vitC** in this study, the ROS-inducing component of this strategy is not limited to small-molecule prooxidants. Previous studies have shown that adoptive transfer of tumor-specific T cells, or infusion of immune checkpoint antibodies, can induce substantial ROS accumulation in tumor cells, contributing to tumor regression in multiple preclinical models [[58], [59], [60], [61], [62]]. Rational combinations of tumor redox-modulating agents with cancer immunotherapy are therefore emerging as promising strategies [63]. The potential combination of our ROS-responsive prodrugs with immunotherapies is currently under investigation.

In summary, our study establishes a novel strategy to unleash the antitumor potential of ROS-responsive prodrugs in a highly tumor-selective manner. By leveraging the synergy between a ROS-inducing prooxidant and a ROS-responsive prodrug, this approach enables robust and selective tumor killing while sparing normal tissues. These findings highlight the therapeutic potential of redox-triggered combination strategies for difficult-to-treat cancers and suggest a new paradigm for repurposing **vitC** in combination therapies to overcome the inconsistent outcomes observed in earlier clinical studies. We recognize that tumor redox states—including basal H_2_O_2_ flux, antioxidant buffering capacity, and labile iron availability—are inherently heterogeneous across tumor types and even within individual tumors, which may influence therapeutic responsiveness. Accordingly, while our mechanistic studies focused on representative breast cancer models (MDA-MB-468, MCF-7, MDA-MB-436, and MDA-MB-231) alongside non-tumorigenic mammary epithelial cells (MCF10A) and primary HMECs, extension of this strategy to additional tumor types and genetic backgrounds will be important to define its broader translational applicability.

### 4.1 Statistical analysis

Data were analyzed using GraphPad Prism 5 (GraphPad Software, Sandiego, CA) and expressed as the mean ± SEM. A student *t*-test was used to find the significance between 2 groups. Comparison of the multiple groups was done using one-way ANOVA analysis. P value of less than 0.05 was considered to be statistically significant. **Power analysis:** Groups of 5 mice are determined with G*Power 3.1 based on the desire to show a significance level of 0.05 and the results of a pilot study.

## Data and materials availability

All data needed to evaluate the conclusions in the paper are present in the paper and/or the Supplementary Materials.

## Funding sources

This work was supported in part by the National Cancer Institute (1R15CA277656-01), UWM Research Foundation Bradley 10.13039/100027448Catalyst Grant Program, UWM Discovery and Innovation Grant, and Great Milwaukee Foundation (Shaw Scientist Award). Alexis Kimberly Peterson was supported in part by UWM Senior Excellence in Research Awards. The undergraduates were also supported in part by UWM Support for Undergraduate Research Fellows (SURF) program.

## CRediT authorship contribution statement

**Taufeeque Ali:** Data curation, Formal analysis, Methodology, Validation, Writing – original draft. **Thilini Nimasha Fernando Ponnamperumage:** Data curation, Formal analysis, Investigation, Methodology, Validation, Visualization, Writing – original draft, Writing – review & editing. **Cody Joshua Miller:** Data curation, Methodology. **Daniel Li:** Data curation, Writing – review & editing. **Wasiu Olaniyi Awoyera:** Data curation, Resources. **Alexis Kimberly Peterson:** Data curation. **Hanlun Gao:** Data curation. **Jatin Pandey:** Data curation. **Julia Anna Rose Jakusz:** Data curation. **Heli Fan:** Data curation. **Gilbert Edward Koelsch:** Data curation. **Leggy A. Arnold:** Methodology, Resources. **Julie M. Jorns:** Formal analysis, Validation. **Yee Chung Cheng:** Conceptualization, Funding acquisition, Resources. **Avik Roy:** Methodology. **Gang Zhou:** Conceptualization, Investigation, Methodology. **Xiaohua Peng:** Conceptualization, Funding acquisition, Investigation, Project administration, Supervision, Validation, Writing – original draft, Writing – review & editing.

## Declaration of competing interest

The authors declare no competing financial interest.

## Data Availability

Data will be made available on request.
