# Supplementary material for: Exploiting vitamin C as a prooxidant to activate ROS-responsive prodrugs for potent and selective tumor killing
Source: Redox Biol. 2026 Jun 4;95:104229. doi: 10.1016/j.redox.2026.104229 (PMC13273229; doi:10.1016/j.redox.2026.104229)
Supplement: Multimedia component 1 [file mmc1.docx]

Supplementary Materials for

**Exploiting vitamin C as a prooxidant to activate ROS-responsive prodrugs for potent and selective tumor killing**

Taufeeque Ali,^a,1^ Thilini Nimasha Fernando Ponnamperumage,^a,1^ Cody Joshua Miller,^a^ Daniel Li,^a^ Wasiu Olaniyi Awoyera,^a^ Alexis Kimberly Peterson,^a^ Hanlun Gao,^a^ Jatin Pandey,^a^ Julia Anna Rose Jakusz,^a^ Heli Fan,^a^ Gilbert Edward Koelsch,^a^ Leggy A. Arnold,^a^ Julie M. Jorns,^b^ Yee Chung Cheng,^c^ Avik Roy,^d^ Gang Zhou,^e^ and Xiaohua Peng^a*^

^a^Department of Chemistry and Biochemistry and the Milwaukee Institute for Drug Discovery, University of Wisconsin-Milwaukee, 2000 E. Kenwood Boulevard, Milwaukee, WI 53211, United States

^b^Department of Pathology, Medical College Wisconsin, Milwaukee, Wisconsin

^c^Department of Hematology and Oncology and Department of Medicine, Medical College Wisconsin, Milwaukee, Wisconsin

^d^Research and Development Laboratory, Simmaron Research Institute, Milwaukee, WI, USA

^e^Georgia Cancer Center, Medical College of Georgia, Augusta University, Augusta, GA, USA

^*^To whom correspondence may be addressed. Email: [pengx@uwm.edu](mailto:pengx@uwm.edu)

^1^ Ali and Ponnamperumage contributed equally to this manuscript.

**This PDF file includes:**

Supplementary Text

Figs S1 to S27

Tables S1 to S11

Supplementary Text

**Safety study with CD-1 mice**. The toxicity of the combination of **vitC** and **FAN-NM-CH_3_** was evaluated with CD-1 mice (fig. S18). **FAN-NM-CH_3_** was administered intraperitoneally (IP) at fixed doses of 5 mg/kg, 10 mg/kg or 20 mg/kg, while the **vitC** dose was escalated starting from 1 g/kg to a maximum of 4 g/kg or until serious adverse events occurred (fig. S18C). A Mouse Intervention Scoring System was employed to monitor mouse health and welfare (Table S8). This system assigned scores based on daily observations of distress, illness, or abnormal behavior, with a terminal score >10 considered normal, 6–9 indicating likely morbidity, and <6 indicating moribund states. Individual mouse scores are plotted in fig. S18G. To assess delayed-onset toxicity before the *in vivo* efficacy study, a repeated-dose toxicity study was conducted over five days (fig. S18 D to F and Table S9). Mice were divided into four groups: one vehicle group and three treatment groups receiving 5, 10, or 20 mg/kg of **FAN-NM-CH_3_** (Group-5, Group-10, and Group-20, respectively; figs. S18 D to F, and figs. S20 and S21). Within each group, fixed **vitC** doses of 500 mg/kg, 750 mg/kg, and 1000 mg/kg were administered IP for five days.

**Fig. S.1.**

**The cytotoxicity dose-response curves.** (**A**) various prooxidants against MDA-MB-468; (**B**) various prooxidants against MCF 10A cell lines; (**C**) Selectivity Index (SI) of prooxidants alone; (D) concentration-dependent cytotoxicity of **FAN-NM-CH_3_** in combination with maximum safe dose of various prooxidants in MDA-MB-468; (**E**) concentration-dependent cytotoxicity of **FAN-NM-CH_3_** in combination with maximum safe dose of various prooxidants in MCF 10A. Cells were incubated for 48 hours post-treatment (n = 3).

**Fig. S.2.**

**The cytotoxicity and selectivity of various prooxidants, FAN-NM-CH_3_ alone or combination therapy on various cancer and normal cells** (**A-F**: data obtained with various cancer and normal cell lines). (**A-B**) The IC_50_ curve and values of vitC alone; (**C**) The IC_50_ values of **FAN-NM-CH_3_** alone or in combination with maxSD of vitC; (**D**) Dose reduction index; **(E)** The effect of maxSD **vitC** on IC_50_ values of **FAN-NM-CH_3_** in various tumor and normal cell lines; **(F)** Combination Index of combination therapy for different tumor and normal cell lines; **(G)** Rescue assay by 100 µM Deferoxamine (DFO) and 7 µM Catalase (CAT); [the data represent three independent experiments performed in triplicate (n = 3)].

**Fig. S3**.

**The drug synergy test for vitC and FAN-NM-CH_3_ by SynergyFinder 3.0 with Dose Response Matrix (left), the drug interaction 2D (middle) and 3D (right) landscapes based on the Bliss model**. The synergy score is shown by Red (> 0) and Green (< 0). A synergy score of < -10 means the interaction is likely to be antagonistic, between -10 to 10 means it’s likely to be additive, and > 10 means it is likely to be synergistic. Tests were performed on data generated from cytotoxicity studies on MCF7 cells when treated for 48 h and data was normalized with their respective untreated controls. All cytotoxicity data used for the synergy score represents 3 independent replicate experiments, mean ± SD (n = 3).

**Fig. S4.**

**H_2_O_2_ Determination**. (**A**) H_2_O_2_ standard curve and the quenching effect of catalase; (**B**) **vitC**-induced H_2_O_2_ generation in KRPG buffer and the quenching effect of catalase on **vitC**-induced H_2_O_2_ formation.

**Fig. S5.**

**PEG-catalase-mediated intracellular H₂O₂ quenching in MDA-MB-468 and MCF 10A cells.** Cells were pre-treated overnight with 160 nM PEG-catalase (Sigma-Aldrich, C4963). After 12 hours, cells were washed with KRPG buffer and replenished with fresh KRPG before treatment with VitC (maxSD). One hour after **VitC** exposure, KRPG was removed and replaced with fresh culture medium. Cells were then treated with FAN-NM-CH₃ (maxSD) and incubated at 37 °C for 48 hours. Cell viability was assessed using CellTiter-Glo. Data represents three independent experiments with triplicate samples (n = 3).

**Fig. S6.**

**Extracellular does-dependent H_2_O_2_ generation induced by vitC in various cell lines**. (**A**) H_2_O_2_ level indicated by fluorescence intensity (Cancer cell lines, MDA-MB-468, MCF7, and normal cell line MCF 10A were treated with various concentrations of **vitC** (0.005-10 mM) and incubated at 37 ℃ for 48 h. Media was replaced with KRPG buffer and cells were incubated at 37 ℃ for 5 h followed by fluorescence measurement); (**B**) Data represents Fluorescence Intensity/Cell (Data were normalized to the number of viable cells); (**C**) Extracellular H_2_O_2_ standard curve (Control H_2_O_2_ samples were prepared in KRPG buffer and incubated for 30 minutes. 20 µL of the KRPG buffer was transferred into a black 96-well plate and incubated with equal volume of 50 µM Amplex Red solution for 5 h at 37 ℃). Fluorescence was measured (Ex/Em: 560/590 nm) on an infinite M1000 (Tecan) microplate reader. Data represents 3 independent experiments with three replicates (n = 3).

**Fig. S7.**

**Intracellular does-dependent H_2_O_2_ generation induced by vitC in various cell lines.** (**A**) H_2_O_2_ level indicated by fluorescence intensity (Cancer cell lines, MDA-MB-468, MCF7, and normal cell line MCF 10A were treated with various concentrations of **vitC** (0.005-10 mM) and incubated at 37 ℃ for 48 h. Media was replaced with KRPG buffer and cells were incubated at 37 ℃ for 5 h followed by fluorescence measurement); (**B**) Data represents Fluorescence Intensity/Cell (Data were normalized to the number of viable cells); (**C**) Intracellular H_2_O_2_ standard curve (Control H_2_O_2_ samples were prepared in KRPG buffer and incubated with cells in the dark at 37 ℃ for 30 mins. All samples were incubated with 1X AbGreen Stain at 37 ℃ for 1 h. Fluorescence was measured (Ex/Em: 490/520) on an infinite M1000 (Tecan) microplate reader. Data represents 3 independent experiments with three replicates (n = 3).

**Fig. S8.**

**Determination of H_2_O_2_ levels induced by safe doses of vitC alone, prodrug alone, or combination of safe doses of vitC and prodrug in various cell lines. (**A**)** Extracellular H_2_O_2_ level indicated by fluorescence intensity; **B**) Data of extracellular H_2_O_2_ level was normalized to the number of viable cells [Cancer cell lines, MDA-MB-468, MCF7, and normal cell line MCF 10A were treated under different conditions and incubated at 37 ℃ for 48 h. Media was replaced with KRPG buffer and cells were incubated at 37 ℃ for 5 h. 20 µL of the KRPG buffer was transferred into a black 96-well plate and incubated with equal volume of 50 µM Amplex Red solution at 37 ℃ for 5 h. Fluorescence was measured (Ex/Em: 560/590 nm) on an infinite M1000 (Tecan) microplate reader. Data represents 3 independent experiments with three replicates (n = 3)]. (**C**) Intracellular H_2_O_2_ level indicated by fluorescence intensity; **D**) Data of intracellular H_2_O_2_ level was normalized to the number of viable cells) [Cells were treated under different conditions and incubated at 37 ℃ for 48 h, washed with KRPG buffer, and incubated with 40 uL of 1X AbGreen stain in the dark at 37 ℃ for 1 h. Fluorescence was measured (Ex/Em: 490/520) on an infinite M1000 (Tecan) microplate reader. Data represents 3 independent experiments with three replicates (n = 3)].

**Fig. S9.**

**Catalase standard curve**. Catalase standards from Catalase Colorimetric Activity kit (Invitrogen: EIACATC) were prepared by diluting 10 µL of the catalase standard solution in 190 µL of 1X assay buffer to obtain a 5 U/mL catalase solution. This solution was further diluted 2-fold five times, including a blank with 1X assay buffer. 25 µL of the standard samples were added to the clear 96-well half-area plate provided with the kit, followed by 25 µL of Hydrogen Peroxide Reagent into each well. The plate was incubated for 30 minutes at room temperature. Subsequently, 25 µL of substrate and 25 µL of 1X HRP solution were added to each well. The plate was incubated at room temperature for 15 minutes. Absorbance was recorded at 560 nm using the Infinite M1000 (Tecan) plate reader. A standard curve was generated using curve-fitting on GraphPad Prism software, and the activity of unknown samples was calculated from the standard curve and adjusted for the appropriate dilution factor.

**Fig. S10**.

**Determination of catalase concentrations. Catalase for H₂O₂ quenching was obtained from Sigma-Aldrich (C3155-100MG)**. To determine the catalase concentration used in the experiments, a one-fourth dilution of the original stock was prepared, and its absorbance was measured using a UV-Vis spectrophotometer. The stock concentration was calculated using a molar extinction coefficient of 420,000 M⁻¹cm⁻¹. For the experiments, a 10% solution (0.722 µM) of the catalase stock was prepared, and 4 µL of this solution was added to each quenching reaction with a final volume of 40 µL, resulting in a final catalase concentration of 0.07 µM.

**Fig. S11.**

**The cytotoxicity dose-response curve of catalase**. To assess catalase toxicity and identify a safe dose, MDA-MB-468 cells were treated with varying concentrations of catalase (Sigma-Aldrich) at 37°C for 48 hrs.

**Fig. S12.**

**Cell viability upon treatment with varying concentrations of vitC in combination with a 5 µM prodrug, in the presence or absence of catalase**. The cells were incubated at 37°C for 48 hrs. Data represents three independent experiments with at least three replicates each (n = 3).

**Fig. S13.**

**Cell viability upon treatment with varying concentrations of vitC in combination with a 3 µM prodrug, in the presence or absence of catalase**. The cells were incubated at 37°C for 48 hrs. Data represents three independent experiments with at least three replicates each (n = 3).

**Fig. S14.**

**Cell viability upon treatment with varying concentrations of vitC in combination with a 2 µM prodrug, in the presence or absence of catalase**. The cells were incubated at 37°C for 48 hrs. Data represents three independent experiments with at least three replicates each (n = 3).

**Fig. S15.**

**Cell viability upon treatment with varying concentrations of vitC in combination with a 1 µM prodrug, in the presence or absence of catalase**. The cells were incubated at 37°C for 48 hrs. Data represents three independent experiments with at least three replicates each (n = 3).

**Fig. S16.**

**Cell viability upon treatment with varying concentrations of vitC in combination with a 0.5 µM prodrug, in the presence or absence of catalase**. The cells were incubated at 37°C for 48 hrs. Data represents three independent experiments with at least three replicates each (n = 3).

**Fig. S17.**

**Quantitative analysis of the comet images using TriTek CometScore Software.** **A**) Intact DNA in the comet heads; **B)** Damaged DNA in the comet tails; **C**) Quantification of the Tail Moment; **D**) Quantification of the Tail Olive Moment.

**Fig. S18**. ***In vivo* safety study**. (**A**) Single dose toxicity study schedule; (**B**) Repeated-dose toxicity study schedule; (**C**) The percentage of mice weight change (%) observed during 7-day period of dose-escalating **vitC** in combination with 5 mg/kg, 10 mg/kg, or 20 mg/kg of prodrug; (**D**) Mice weight change during 5-day treatment and 2 days recovery with 500 mg/kg, 750 mg/kg, or 1 g/kg dose of **vitC** in combination with 5 mg/kg of prodrug; (**E**) Mice weight change during 5-day treatment and 2 days recovery with 500 mg/kg, 750 mg/kg, or 1 g/kg dose of **vitC** in combination with 10 mg/kg of prodrug; (**F**) Mice weight change during 5-day treatment and 2 days recovery with 500 mg/kg, 750 mg/kg, and 1 g/kg dose of **vitC** in combination with 20 mg/kg of prodrug; (**G**) Mouse Intervention Scores for single dose toxicity study; and (**H**) Mouse Intervention Scores for repeated-dose studies. For each study, mice were divided into four groups, each containing three mice.

**Fig. S19.**

Changes of mice body weight observed during a 9-day period of dose-escalating **vitC** in combination with prodrug (n = 3). The mice were divided into four groups, each containing three mice.

(**A**)

(**B**)

**Fig. S20.**

**Repeat dose toxicity study**. (**A**) Changes of mice body weight during 5-day treatment and 2 days post-treatment observation with 500 mg/kg, 750 mg/kg, and 1 g/kg doses of **vitC** in combination with 5 mg/kg of prodrug (n =3); (B) Changes of mice body weight during 5-day treatment and 2 days post-treatment observation with 500 mg/kg, 750 mg/kg, and 1 g/kg doses of **vitC** in combination with 10 mg/kg of prodrug (n =3). The mice were divided into four groups, each containing three mice.

**Fig. S21.**

**Repeat dose toxicity study**. Changes of mice body weight during 5-day treatment and 2 days post-treatment observation with 500 mg/kg, 750 mg/kg, and 1 g/kg doses of **vitC** in combination with 20 mg/kg of prodrug (n =3). The mice were divided into four groups, each containing three mice.

**Fig. S22.**

**In vivo efficacy study**. (**A**) Time-dependent tumor growth measured by caliper during xenograft study involving four groups: vehicle, **vitC**, prodrug, and combination (n = 5); (**B**) Representative mice picture before and after treatment. The mice received intraperitoneal (IP) administrations of vehicle, 500 mg/kg **vitC**, 3 mg/kg prodrug, and sequential doses of 500 mg/kg **vitC** followed by 3 mg/kg prodrug after 1 hour in the combination group.

**Fig. S23.**

**Body weight change for the xenograft mice treated with vehicle, vitC, prodrug, and combination (n = 5).** The mice received intraperitoneal (IP) administrations of vehicle, 500 mg/kg **vitC**, 3 mg/kg prodrug, and sequential doses of 500 mg/kg **vitC** followed by 3 mg/kg prodrug after 1 h in the combination group.

**Fig. S24.**

**Post-treatment effects and toxicity**. (A) Mice Weight (g) (B) % Mice weight change (g) (C) Tumor volume (mm^3^) and (D) % Tumor volume change (mm^3^).

**Fig. S25.**

**In vivo efficacy and safety**. (**A**) Appearance of mice with tumors at the end of week-10 treatment; (**B**) Appearance of organs of dissected mice at the end of week-10 treatment; (**C**) Mouse Intervention Scores for Efficacy study.

|  | Appearance of mice | H&E Stained Organs and Tumors. | | | | | | |
| --- | --- | --- | --- | --- | --- | --- | --- | --- |
|  |  | Heart | Liver | Spleen | Lung | Kidney | Brain | Tumor |
| Vehicle |  |  |  |  |  |  |  |  |
| **vitC** |  |  |  |  |  |  |  |  |
| **Prodrug** |  |  |  |  |  |  |  |  |
| Comb. |  |  |  |  |  |  |  |  |
|  |  |  |  |  |  |  |  |  |

**Fig. S26.**

**The safety of vitC, low doses of prodrug, and combination therapy with mice organs**. No alteration in appearance of mice organs after 10-weeks treatment and H&E stain of different organs and tumors (H&E, 20X).

**Fig. S27.**

**Kaplan–Meier Plot.** Survival analysis defining an event as tumor volume exceeding 1000% of its baseline size.

**Table S1.**

Comparison of IC_50_ (µM) of prooxidants and their selectivity towards cancer cells.

| Compounds | IC_50_ (µM) | | | | | IC_50_ of **FAN-NM-CH_3_** (µM)^b^ | | |
| --- | --- | --- | --- | --- | --- | --- | --- | --- |
|  | MDA-MB-468 Cancer Cells | | MCF 10A Normal Cells | | SI^c^ | Cancer Cells | Normal Cells | SI^c^ |
|  | Mean ± SD  (n = 3) | MAXSD^a^ (µM) | Mean ± SD  (n = 3) | MAXSD^a^ (µM) |  |  |  |  |
| **FAN-NM-CH_3_** | 3.02 ± 0.2 | 1 | 48.40 ± 3.1 | 6 | 16 | - | - | - |
| **vitC** | 2330 ± 300 | 1000 | 4200 ± 400 | 2000 | 2 | 0.49 | 35.9 | 73 |
| Piperlogumine | 2.24 ± 0.05 | 0.78 | 4.91 ± 0.51 | 1.56 | 2 | 1.31 | 7.58 | 6 |
| β-Lapachone | 1.06 ± 0.17 | 0.78 | 3.42 ± 0.22 | 1.56 | 3 | 0.73 | 1.28 | 2 |
| Vitamin E | 76.5 ± 6.62 | 1.56 | 375.8 ± 21.4 | 25.00 | 5 | 2.37 | 25.8 | 11 |
| Arsenic Trioxide | 6.27 ± 0.33 | 0.79 | 8.52 ± 0.40 | 1.56 | 1 | 0.97 | 4.99 | 5 |
| Propyl Gallate | 49.23 ± 1.83 | 25.00 | 51.35 ± 5.45 | 12.50 | 1 | 3.24 | 10.6 | 3 |
| Quercetin | 15.83 ± 2.15 | 3.13 | 19.59 ± 1.24 | 6.25 | 1 | 2.22 | 6.07 | 3 |
| EGCG | 64.97 ± 3.45 | 12.50 | 28.51 ± 2.55 | 6.25 | 0.5 | 2.15 | 4.54 | 2 |
| ^a^The Maximum Safe Dose (MAXSD) defined as the highest dose without causing discernible toxicity towards both cancer and normal cells.  ^b^IC_50_ of **FAN-NM-CH_3_** when combined with MAXSD of the prooxidants.  ^c^Selectivity Index defined as the ratio of IC50 value in Normal MCF 10A cells to that in MDA-MB-468 cancer cells. | | | | | | | | |

**Table S2.**

The cytotoxicity of **vitC** in different cell lines.

| **Cell Types** | **Cell lines** | **IC_50_ (mM)^a^** |
| --- | --- | --- |
| Breast Cancer | MCF7 | 0.97 ± 0.07 |
| Triple-negative Breast Cancer | MDA-MB-468 | 2.33 ± 0.3 |
|  | MDA-MB-436 | 1.39 ± 0.1 |
|  | MDA-MB-231 | 2.23 ± 0.3 |
| Normal Epithelial | HMEC | 2.67 ± 0.4 |
|  | MCF10A | 4.20 ± 0.4 |
| ^a^The IC_50_ values of **vitC** in cancerous cells as well as normal cells when incubated at 37 °C for 48 h (n = 3, the IC_50_ values were determined by a nonlinear regression). | | |

**Table S3.**

Cytotoxicity of **FAN-NM-CH_3_** in different cell lines, Maximum Safe Dose of Ascorbic acid, and Cytotoxicity in Combination.

| Cell lines | IC_50_ of **FAN-NM-CH_3_** (µM)^a^ | Maximum Safe Dose of **vitC**^b^ (mM) | IC_50_ of **FAN-NM-CH_3_** in Combination with Safe Dose of **vitC** (µM)^c^ | CI^d^ | DRI 1^e^ | DRI 2^f^ |
| --- | --- | --- | --- | --- | --- | --- |
| MDA-MB-468 | 3.02 ± 0.2 | 1.00 | 0.49 ± 0.05 | 0.59 | 6.2 | 2 |
| MCF7 | 6.60 ± 0.5 | 0.25 | 2.14 ± 0.3 | 0.58 | 3.1 | 4 |
| MDA-MB-231 | 3.45 ± 0.3 | 1.0 | 0.95 ± 0.1 | 0.72 | 3.6 | 2 |
| MDA-MB-436 | 3.65 ± 0.2 | 0.63 | 1.56 ± 0.1 | 0.87 | 2.3 | 1 |
| MDA-MB-468 + Chlorambucil | 30.17 ± 2.3 | 1 | 28.66 ± 2.4 | 1.38 | 1.1 | 2 |
| MCF 10A (normal cells) | 48.01 ± 3.5 | 1.25 | 35.87 ± 2.6 | 1.04 | 1.3 | 3 |
| HMEC | 10.09 ± 0.06 | 0.15 | 26.9 ± 0.3 | 3.2 | 0.37 | 17 |
| ^a^IC_50_ of the prodrug alone when incubated for 48 h (n = 3, the IC_50_ values were determined by a nonlinear regression).  ^b^The Maximum Safe Dose (MAXSD) defined as the highest dose without causing discernible toxicity towards both cancer and normal cells.  ^c^ IC_50_ of the prodrug in combination with MAXSD of **vitC** (n = 3, the cells were incubated at 37 °C for 48 h and the IC_50_ values were determined by a nonlinear regression).  ^d^The Combination Index (CI) determines the synergy effect between two drugs: CI < 1 (Synergistic effect), CI = 1 (additive effect), and CI > 1 (antagonistic effect).  ^e,f^Dose Reduction Index (DRI), determines the fold reduction in drug dose when given in combination. A higher DRI value indicates a reduced drug dosage is needed in combination to achieve the same efficacy as a single drug. | | | | | | |

**Table S4.** Intracellular H_2_O_2_ levels assessed using AbGreen indicator in live cells under varied treatments over 48 h of incubation at 37 ℃.

| **Intracellular H_2_O_2_** | **MCF10A^a^** | **MDA-MB-468^b^** | **MCF7^c^** |
| --- | --- | --- | --- |
| Media |  |  |  |
| Cells  (Without Dye) |  |  |  |
| Cells |  |  |  |
| Cells + CAT |  |  |  |
| H_2_O_2_ (100 µM) |  |  |  |
| H_2_O_2_ (100 µM)  +  CAT |  |  |  |
| Cells  +  **vitC** (MAXSD) ^a,b,c^ |  |  |  |
| Cells  +  **vitC** (MAXSD) ^a,b,c^  +  CAT |  |  |  |
| Cells  +  Prodrug^a,b,c^ |  |  |  |
| Cells  +  Prodrug^a,b,c^  +  CAT |  |  |  |
| Cells  +  **vitC** (MAXSD)^a,b,c^  +  Prodrug^a,b,c^ |  |  |  |
| Cells  +  **vitC** (MAXSD)^a,b,c^  +  Prodrug^a,b,c^  +  CAT |  |  |  |
| ^a^ MAXSD of vitC in **MCF10A** is 1.0 mM; 1.0 µM prodrug was used for **MCF10A** cells.  ^b^ MAXSD of vitC in **MDA-MB-468** is 1.0 mM; 1.0 µM prodrug was used for **MDA-MB-468** cells.  ^c^ MAXSD of vitC in **MCF 7** is 0.25 mM; 2.0 µM prodrug was used for **MCF7** cells | | | |

**Table S5.** The alkaline comet assay images of MDA-MB-468, MCF7, and MCF 10A cells after treatment with vehicle, **vitC**, **FAN-NM-CH_3_**, and the combination over 48 h of incubation at 37 ℃.

| **Sample** | **MCF10A^a^** | **MDA-MB-468^b^** | **MCF7^c^** |
| --- | --- | --- | --- |
| Cells |  |  |  |
| Cells  +  CAT |  |  |  |
| H_2_O_2_  (100 µM) |  |  |  |
| H_2_O_2_  (100 µM)  +  CAT |  |  |  |
| Cells  +  **vitC** (MAXSD) ^a,b,c,d^ |  |  |  |
| Cells  +  **vitC** (MAXSD)^a,b,c,d^  +  CAT |  |  |  |
| Cells  +  Prodrug^a,b,c,d^ |  |  |  |
| Cells  +  Prodrug^a,b,c,d^  +  CAT |  |  |  |
| Cells  +  **vitC** (MAXSD)^a,b,c,d^  +  Prodrug^a,b,c,d^ |  |  |  |
| Cells  +  **vitC** (MAXSD)^a,b,c,d^  +  Prodrug^a,b,c,d^  +  CAT |  |  |  |
| Cells  +  10 mM **vitC** |  |  |  |

**Table S6.** Images of cell’s DNA damage assessed by TriTek CometScore Software.

|  | Cells | CAT | Prodrug | Prodrug + CAT | VitC | VitC + CAT | VitC + Prodrug | VitC + Prodrug + CAT |
| --- | --- | --- | --- | --- | --- | --- | --- | --- |
| MDA-MB-468 |  |  |  |  |  |  |  |  |
| MCF7 |  |  |  |  |  |  |  |  |
| MCF10A |  |  |  |  |  |  |  |  |

**Table S7.** The images of Annexin V/PI/DAPI stained cells under varied treatments.

| MDA-MB-468 | DAPI | Annexin V | PI | Merge | Magnified |
| --- | --- | --- | --- | --- | --- |
| Vehicle |  |  |  |  |  |
| **vitC**  (1 mM) |  |  |  |  |  |
| Prodrug  (1 µM) |  |  |  |  |  |
| Comb. |  |  |  |  |  |

| MCF7 | DAPI | Annexin V | PI | Merge | Magnified |
| --- | --- | --- | --- | --- | --- |
| Vehicle |  |  |  |  |  |
| **vitC**  (0.25 mM) |  |  |  |  |  |
| Prodrug  (2 µM) |  |  |  |  |  |
| Comb. |  |  |  |  |  |

| MCF 10A | DAPI | Annexin V | PI | Merge | Magnified |
| --- | --- | --- | --- | --- | --- |
| Vehicle |  |  |  |  |  |
| **vitC**  (1 mM) |  |  |  |  |  |
| Prodrug  (1 µM) |  |  |  |  |  |
| Comb. |  |  |  |  |  |

**Table S8.** Mice Scoring Chart

| **Parameter** | **Description** | **Score** |
| --- | --- | --- |
| Appearance | Normal (smooth coat, clear eyes/nose) | 2 |
|  | Hunched and/or fluffed | 1 |
|  | Ocular discharge and/or edema | 0 |
| Natural behavior | Active; interactive in environment | 3 |
|  | Less interactive | 2 |
|  | Isolated; pronounced decrease in activity | 1 |
|  | Immobile | 0 |
| Provoked behavior | Normal (quickly moves when cage is disturbed) | 3 |
|  | Subdued; (moves away slowly) | 2 |
|  | Subdued even to stimulation (moves away after short period of time) | 1 |
|  | Unresponsive to gentle podding | 0 |
| Weight loss | < 20% | 3 |
|  | 20-25% | 2 |
|  | 26-30% | 1 |
|  | >30% | 0 |
| Total score | Normal | > 10 |
|  | Morbid; monitor atleast 3 times per day | 9 to 6 |
|  | Moribund; euthanize immediately, consider as found dead | < 6 |

**Table S9**. Mouse Survival Rate after Treatment

|  | Single Dose | | | | Repeated Dose | | |
| --- | --- | --- | --- | --- | --- | --- | --- |
| Compounds | **vitC** (g/kg) | | | | **vitC** (mg/kg) | | |
| Prodrug (mg/kg) | 1 | 2 | 3 | 4 | 500 | 750 | 1000 |
| 5 | 3/3 | 3/3 | 3/3 | 3/3 | 3/3 | 3/3 | 3/3 |
| 10 | 3/3 | 3/3 | 3/3 | 3/3 | 3/3 | 3/3 | 3/3 |
| 20 | 3/3^a^ | 3/3^a^ | 1/3^a^ | n.d | 3/3^a^ | 3/3^a^ | 3/3^a^ |
| ^a^Symptoms of toxicity were observed, including weight loss, loss of appetite, and reduced activity levels. | | | | | | | |

**Table S10.** Organs harvested from MDA-MB-468 xenografted mice after 10 weeks of treatment.

| Vehicle Group | **vitC** Group |
| --- | --- |
|  |  |
| **FAN-NM-CH_3_** Group | Combination Group |
|  |  |

**Table S11.** H&E-stained organs and tumors.

|  | Vehicle | **vitC** (500 mg/kg) | Prodrug (3 mg/kg) | Combination |
| --- | --- | --- | --- | --- |
| Heart (Full) |  |  |  |  |
| Heart (20X) |  |  |  |  |
| Liver (Full) |  |  |  |  |
| Liver (20X) |  |  |  |  |
| Spleen (Full) |  |  |  |  |
| Spleen (20X) |  |  |  |  |
| Lung (Full) |  |  |  |  |
| Lungs (20X) |  |  |  |  |
| Kidney (Full) |  |  |  |  |
| Kidney (20X) |  |  |  |  |
| Brain (Full) |  |  |  |  |
| Brain (20X) |  |  |  |  |
| Tumor (Full) |  |  |  |  |
| Tumor (20X) |  |  |  |  |
